# The chromatin remodeling factor CHD7 controls cerebellar development by regulating reelin expression

**DOI:** 10.1172/JCI83408

**Published:** 2017-02-06

**Authors:** Danielle E. Whittaker, Kimberley L.H. Riegman, Sahrunizam Kasah, Conor Mohan, Tian Yu, Blanca Pijuan Sala, Husam Hebaishi, Angela Caruso, Ana Claudia Marques, Caterina Michetti, María Eugenia Sanz Smachetti, Apar Shah, Mara Sabbioni, Omer Kulhanci, Wee-Wei Tee, Danny Reinberg, Maria Luisa Scattoni, Holger Volk, Imelda McGonnell, Fiona C. Wardle, Cathy Fernandes, M. Albert Basson

**Affiliations:** 1 King’s College London, Department of Craniofacial Development and Stem Cell Biology, Guy’s Hospital Tower Wing,; 2 Department of Comparative Biomedical Sciences, Royal Veterinary College, and; 3 King’s College London, Randall Division, New Hunt’s House, London, United Kingdom.; 4 Neurotoxicology and Neuroendocrinology Section, Department of Cell Biology and Neuroscience, Istituto Superiore di Sanità, and; 5 School of Behavioural Neuroscience, Department of Psychology, Sapienza University of Rome, Rome, Italy.; 6 Department of Physiology, Anatomy and Genetics, University of Oxford, Oxford, United Kingdom.; 7 Department of Physiology and Pharmacology “V. Erspamer,” Sapienza University of Rome, Rome, Italy.; 8 MRC Social, Genetic & Developmental Psychiatry Centre, Institute of Psychiatry, Psychology & Neuroscience, King’s College London, London, United Kingdom.; 9 Howard Hughes Medical Institute, Department of Molecular Pharmacology and Biochemistry, New York University School of Medicine, New York, New York, USA.; 10 King’s College London, MRC Centre for Neurodevelopmental Disorders, New Hunt’s House, London, United Kingdom.

## Abstract

The mechanisms underlying the neurodevelopmental deficits associated with CHARGE syndrome, which include cerebellar hypoplasia, developmental delay, coordination problems, and autistic features, have not been identified. CHARGE syndrome has been associated with mutations in the gene encoding the ATP-dependent chromatin remodeler CHD7. CHD7 is expressed in neural stem and progenitor cells, but its role in neurogenesis during brain development remains unknown. Here we have shown that deletion of *Chd7* from cerebellar granule cell progenitors (GCps) results in reduced GCp proliferation, cerebellar hypoplasia, developmental delay, and motor deficits in mice. Genome-wide expression profiling revealed downregulated expression of the gene encoding the glycoprotein reelin (*Reln*) in *Chd7*-deficient GCps. Recessive *RELN* mutations have been associated with severe cerebellar hypoplasia in humans. We found molecular and genetic evidence that reductions in *Reln* expression contribute to GCp proliferative defects and cerebellar hypoplasia in GCp-specific *Chd7* mouse mutants. Finally, we showed that CHD7 is necessary for maintaining an open, accessible chromatin state at the *Reln* locus. Taken together, this study shows that *Reln* gene expression is regulated by chromatin remodeling, identifies CHD7 as a previously unrecognized upstream regulator of *Reln*, and provides direct in vivo evidence that a mammalian CHD protein can control brain development by modulating chromatin accessibility in neuronal progenitors.

## Introduction

Mutations in genes encoding chromatin remodeling factors have emerged as a major cause of neurodevelopmental disorders (1). The mechanisms employed by these factors to ensure precise regulation of developmental gene expression remain largely unexplored. Furthermore, the neuroanatomical abnormalities that result from these mutations and the behavioral and psychiatric features associated with specific brain defects remain unidentified for most of these conditions. Chromodomain–helicase–DNA-binding (CHD) proteins are ATP-dependent chromatin remodeling factors that use ATP to catalyze nucleosome translocation along chromatin, presumably to modulate access of transcriptional regulators (2). Mutations in *CHD7* cause CHARGE syndrome, a complex developmental syndrome defined by a constellation of birth defects, which include coloboma, heart defects, atresia of the choanae, retarded growth and development, and genital and ear abnormalities (3–5). Although neurodevelopmental abnormalities are not considered for clinical diagnosis, 99% of patients exhibit developmental delay and 74% suffer from intellectual disability (6). The alterations in chromatin and brain structure that underlie these deficits have not been identified.

ChIP sequencing (ChIP-Seq) experiments in a variety of cell lines have identified widespread CHD7 recruitment across the genome (7–9). CHD7 appears to localize primarily to distal regulatory elements marked by lysine 4 monomethylation on histone 3 (H3K4me1). In vitro nucleosome remodeling assays have confirmed the ability of CHD7 to translocate nucleosomes along a chromatin template and demonstrated a loss or reduction in this activity in CHARGE syndrome–associated CHD7 mutants (10). These findings point to chromatin remodeling defects as a central pathogenic mechanism in CHARGE syndrome. However, whether this holds true in vivo and the effect of such changes on developmental gene expression have not been investigated.

As a first step toward identifying the neuroanatomical alterations that may underlie the neurological deficits in CHARGE syndrome, we recently reported cerebellar hypoplasia in 35% of patients with *CHD7* mutations (11). It remains unknown to what extent cerebellar defects contribute to the developmental delay and intellectual disability associated with CHARGE syndrome.

The cerebellum develops over several weeks in mice and several months in humans. The cerebellar territory is established during early embryogenesis by the action of secreted signaling molecules from a secondary organizer located at the mid-hindbrain boundary (reviewed in ref. 12). We previously reported that biallelic expression of *Chd7* was essential for maintaining appropriate levels of *Fgf8* expression at this early stage of cerebellar development (11). As deregulated *Fgf8* expression and signaling from this embryonic organizer selectively disrupt the formation of the cerebellar vermis, these findings identified a role for CHD7 in early embryonic cerebellar development (13, 14). Toward the end of embryogenesis, the cerebellum initiates a period of rapid growth, primarily driven by the proliferation of granule cell progenitors (GCps) in the external granule layer (EGL) on the surface of the cerebellar anlage. The primary mitogen driving GCp proliferation is Sonic Hedgehog (SHH), which is produced by postmigratory Purkinje cells (PCs) that become organized in a layer beneath the EGL during late embryonic stages (15–17). This process is associated with the formation of cerebellar folia. Intriguingly, we have also detected cerebellar foliation anomalies in CHARGE syndrome patients (11), implying a role for CHD7 in GCp development.

Several genes associated with cerebellar hypoplasia, developmental delay, ataxia, and intellectual disability in humans are expressed in cerebellar GCps (reviewed in ref. 18). Homozygous mutations in *RELN*, which encodes the secreted glycoprotein reelin, or the gene encoding its receptor *VLDLR* are responsible for severe cerebellar hypoplasia and intellectual disability in humans (19, 20). Studies in mouse models have localized *Reln* expression to GCps and have reported essential roles for RELN signaling in PC migration, maturation, and GCp proliferation (21–23). *RELN* has also been associated with psychiatric disease in several contexts. For example, RELN expression has been reported to be reduced in postnatal cerebella from autism patients (24, 25).

In this article, we report a role for CHD7 in controlling GCp proliferation and survival, and a striking downregulation of *Reln* gene expression in *Chd7*-deficient GCps. Increasing *Reln* expression partially rescued GCp proliferation and cerebellar hypoplasia, providing functional genetic evidence that *Reln* downregulation contributes to the cerebellar hypoplasia phenotype. Finally, we show that DNA accessibility is reduced at the *Reln* locus, and throughout the genome in CHD7-deficient GCps, consistent with a role for CHD7 in maintaining DNA accessibility through nucleosome remodeling.

## Results

### Chd7 deletion from neuronal progenitors results in cerebellar hypoplasia.

**Figure 1:**
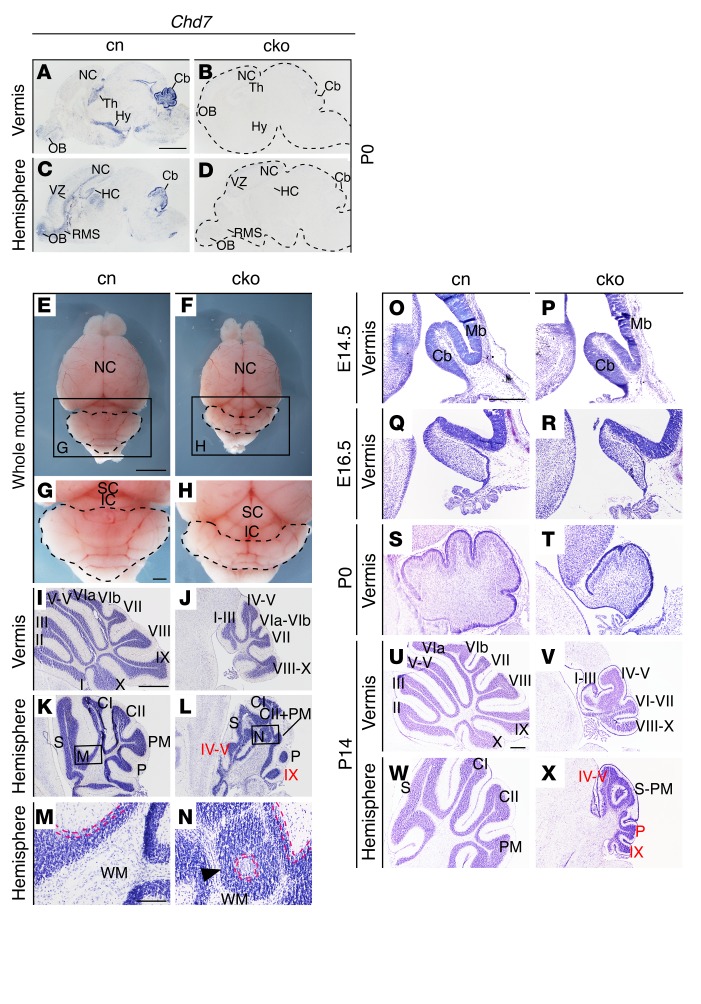
*Chd7* deletion from embryonic neural progenitors causes cerebellar hypoplasia and foliation abnormalities. (**A**–**D**) In situ hybridization for *Chd7* exon 3 transcripts (blue) in sagittal sections through P0 *Chd7^fl/fl^* (control [cn]) and nestin-*Cre Chd7^fl/fl^* (cko) brains. Sections through the cerebellar vermis (**A** and **B**) and hemispheres (**C** and **D**) are shown. Note *Chd7* expression in the olfactory bulb (OB), neocortex (NC), thalamus (Th), hypothalamus (Hy), hippocampus (HC), ventricular zone (VZ), rostral migratory stream (RMS), and cerebellum (Cb) in cn sections (**A** and **C**), absent in the cko (**B** and **D**). (**E** and **F**) Whole-mount images (anterior to the top) with cerebellum outlined. (**G** and **H**) High-power images with cerebella outlined. SC, superior colliculus; IC, inferior colliculus. (**I**–**N**) Cresyl violet–stained sagittal sections through the cerebellum, anterior to the left, with vermis folia labeled with Roman numerals according to Inouye and Oda (77). The simplex (S), Crus I (CI), Crus II (CII), paramedian (PM), and pyramidis (P) folia are labeled in hemisphere sections. Note cerebellar hypoplasia (**J** and **L**) and disorganized folia in cko hemispheres (**L**, boxed area). Note the expansion of lobules IV–V and IX from vermis into the hemispheres (**L**, red text). (**M** and **N**) Magnified view of the boxed areas in **K** and **L** with PC layers outlined in broken red lines. Ectopic granule cells organized around a circular cluster of PCs are indicated with a black arrowhead. WM, white matter. (**O**–**X**) Cresyl violet–stained sections through the developing cerebellum at the time points indicated, anterior to the left, with cerebellar folia labeled as above. Note the vermis hypoplasia at E16.5 and P0, and striking hypoplasia of both vermis and hemispheres at P14 (**V** and **X**) in the cko. Mb, midbrain. Scale bars: 1 mm (**A**, **E**, **I**, and **O**), 300 μm (**G** and **U**), 100 μm (**M**).

*Chd7* is expressed in neural stem cells, where it has been reported to regulate the expression of developmental and disease-associated genes (7). *Chd7* expression localizes to regions of ongoing neurogenesis in the developing brain: the ventricular zone (VZ) and hippocampus (Figure 1, A and C), as well as the rhombic lip stream, VZ, and EGL of the cerebellum (Supplemental Figure 1, A–E; supplemental material available online with this article; doi:10.1172/JCI83408DS1). *Chd7* expression is maintained in postmitotic, differentiating, and migrating neural progenitors in the rostral migratory stream of the forebrain, and upregulated in the differentiating inner EGL and internal granule cell layer of the cerebellum (Supplemental Figure 1E and Figure 1, A and C), implicating *Chd7* in neural differentiation. Several recent studies have identified roles for *Chd7* in adult neural stem cell populations in the forebrain (26–28); yet *Chd7* appears to have a relatively minor role in regulating neural stem cell expansion in the embryonic and perinatal forebrain (26). To determine whether *Chd7* has a function in embryonic neurogenesis, we inactivated *Chd7* in neuronal progenitors using a nestin-*Cre* line (29). In agreement with previous demonstrations of robust gene recombination in the cerebellar anlage by E11 (14), *Chd7* mRNA was not detectable in the nestin-*Cre Chd7^fl/fl^* conditional knockout (cko) cerebellum at E12.5 (Supplemental Figure 1, F and G), and CHD7 protein, present in cell nuclei in the VZ and migratory cells emerging from the rhombic lip in control embryos, was absent from these cells (Supplemental Figure 1, H and I). The expression of *Fgf8* from the mid-hindbrain organizer was maintained and the expression patterns of the *Otx2* and *Gbx2* homeobox genes were not altered in these mutants (Supplemental Figure 1, J–O), indicating that the deletion of *Chd7* after the establishment of this embryonic organizer had little effect on the expression of genes linked to organizer function (11).

Newborn cko animals lacked *Chd7* expression throughout the entire brain (Figure 1, B and D). These mice were born at the expected Mendelian ratios, but postnatal survival rate was reduced (Supplemental Table 1). Two mutants survived to P21; these were smaller than their littermates, overall brain size was reduced accordingly, but cerebellar size was disproportionally reduced (compare cerebella outlined in Figure 1, E with F, and G with H). Histological examination confirmed pronounced hypoplasia of all cerebellar lobules in the vermis (Figure 1, I and J). The cerebellar hemispheres were also hypoplastic and displayed highly irregular cerebellar foliation with apparently misdirected folia (Figure 1, K and L) and ectopic clusters of granule cells around clusters of PCs (Figure 1N). Following specific folia in serial sections (Supplemental Figure 2) revealed that vermis folia IV–V and IX extended abnormally into the lateral hemispheres (Supplemental Figure 2, I and J).

To identify the developmental stage at which these phenotypes first emerge, sagittal sections from E12.5 to P14 cerebella were examined. Cerebellar size was normal at E12.5 (Supplemental Figure 1, F–I) and E14.5 (Figure 1, O and P). Clear cerebellar growth retardation became evident at E16.5 (Figure 1, Q and R), a time point shortly after the initiation of GCp proliferation. At birth (P0), cerebellar hypoplasia was most clearly present in the cerebellar vermis, where it was associated with a failure to initiate the formation of most of the cardinal cerebellar fissures (Figure 1, S and T). By P14, the cerebellum was reduced in size, with irregular foliation that included expansion of vermis lobules IV–V and IX into the hemispheres (Figure 1, U–X). Taken together, these experiments identified a critical role for *Chd7* in cerebellar growth after E14.5, implying a function for CHD7 in the expansion of cerebellar GCps.

### CHD7 regulates the proliferation, differentiation, and survival of cerebellar GCps.

To specifically determine the function of *Chd7* within the granule cell lineage, we deleted *Chd7* from these cells from the time of their specification in the rhombic lip using a *Math1-Cre* transgene (30). Transgenic expression resulted in the recombination of the *Chd7* conditional allele from the early stages of EGL formation at E14.5 (Supplemental Figure 3, C and D). By postnatal stages, efficient *Chd7* deletion was evident in GCps in the anterior and central cerebellar vermis, with *Chd7* expression reduced somewhat in anterior lobule VIII and spared in the most posterior (posterior IX and X) lobules, in agreement with previous reports on the activity of this transgene (Supplemental Figure 3, E–H, and ref. 31). Efficient *Chd7* deletion was evident throughout the entire cerebellar hemispheres (Supplemental Figure 3, I and J). Immunostaining showed the same pattern of CHD7 protein depletion in the cerebellar vermis (Supplemental Figure 3, K–R). Together, these experiments identified the granule cell lineage as the predominant cell type in the postnatal cerebellum that express CHD7, with only faint expression remaining in cells in the white matter after granule cell–specific *Chd7* deletion (Supplemental Figure 3, H and J). Efficient *Chd7* deletion in GCps was further confirmed by quantitative reverse transcriptase PCR (RT-PCR) analysis of purified GCps from P7 animals (Supplemental Figure 3S).

**Figure 2:**
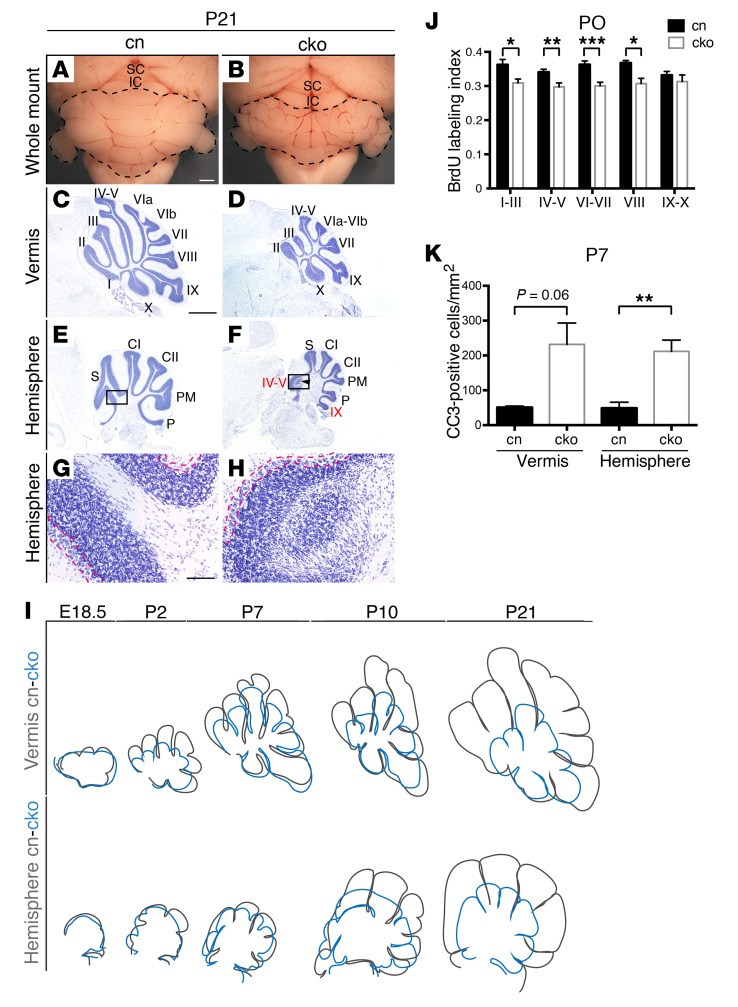
*Chd7* regulates the proliferation and survival of cerebellar GCps. (**A** and **B**) Dorsal whole-mount images of P21 *Chd7^fl/fl^* cn and *Math1-Cre Chd7^fl/fl^* GCp-specific conditional knockout (cko) mouse cerebella, anterior to the top. Note hypoplasia and abnormal foliation in the cko. (**C**–**F**) Cresyl violet–stained sagittal sections through the cerebellum, anterior to the left. Folia are numbered as in Figure 1. Note hypoplasia and abnormal folia indicated by an arrowhead and labeled in red in the cko. (**G** and **H**) Magnified view of the boxed areas in **E** and **F** with PC layers outlined by red broken lines. (**I**) Traced outlines of representative cerebellar sections from cn (black) and cko (blue) mice at the indicated time points. Foliation defects are first noted at E18.5 in the vermis and P2 in the hemispheres. Hypoplasia becomes evident in the vermis by E18.5 and hemispheres by P10 (see also Supplemental Figure 5). (**J**) Quantification of GCp proliferation in the P0 EGL by BrdU incorporation within a 1-hour time window. The BrdU labeling index for the indicated regions representing the fraction of BrdU-positive GCps in the EGL as counted in 100-by-100-μm areas. (**K**) The number of cleaved caspase-3–positive (CC3-positive) cells in the EGL per square millimeter of granule cell area at P7 is shown for vermis and hemispheres (*n* = 3 per genotype). *P < 0.05, **P < 0.01, ***P < 0.001, Student’s *t* test. Scale bars: 300 μm (**A**), 1 mm (**C**), 100 μm (**G**).

The GCp-specific conditional mutants, from here on referred to as cko animals, survived to adulthood. Examination of whole-mount P21 cerebella revealed significant cerebellar hypoplasia (Figure 2, A and B). Histological sections confirmed hypoplasia of lobules I–VIII (Figure 2, C and D), with the most striking hypoplasia presenting in central lobules. All lobules in the hemispheres were smaller, and vermis folia IV–V and IX were again found to extend abnormally into the hemispheres (Supplemental Figure 4 and Figure 2, E and F) with associated foliation irregularities (Figure 2, G and H). Cerebellar development was followed from E13.5 to reveal the first signs of reduced cerebellar growth. Surface area measurements to estimate cerebellar size were performed on Cresyl violet–stained sagittal sections through the most medial aspect of the cerebellar vermis. Delayed initiation of cerebellar foliation in the vermis became evident at E17.5 (Supplemental Figure 5) before cerebellar size was significantly altered (mean area ± SEM = 0.26 ± 0.068 mm^2^ in controls, 0.26 ± 0.026 mm^2^ in cko). Hypoplasia became evident at E18.5 (mean ± SEM = 0.50 ± 0.014 mm^2^ in controls, 0.30 ± 0.026 mm^2^ in cko). To visualize the trajectory of cerebellar growth, representative sections (vermis and hemisphere) from control and cko cerebella were traced and overlaid (Figure 2I). This analysis revealed interesting differences: whereas vermis growth was clearly reduced from E18.5 onward, hypoplasia of the hemispheres became obvious only from P7 (Figure 2I).

To determine the underlying cause of cerebellar hypoplasia, GCp proliferation was quantified by BrdU incorporation between E18.5 and P14. This analysis revealed a significant reduction in GCp proliferation in vermis lobules I–VIII at early postnatal stages (Supplemental Figure 6, A and B), most prominent at P0 (Figure 2J). No significant reduction in GCp proliferation was seen in hemispheres (Supplemental Figure 6D). These results indicated that reduced GCp proliferation likely contributed to reduced growth of the vermis, but not the hemispheres. To assess the contribution of apoptosis, sections were stained with an antibody against activated cleaved caspase-3. The number of apoptotic GCps was increased in both vermis and hemispheres at P7 (Supplemental Figure 6, E and F), but reached statistical significance only in the hemispheres (Figure 2K). These data suggested that the reduced postnatal growth of the hemispheres was largely caused by increased cell death, whereas both proliferative and apoptotic changes contributed to vermis hypoplasia.

### Reduced PC numbers in Chd7 cko cerebella.

**Figure 3:**
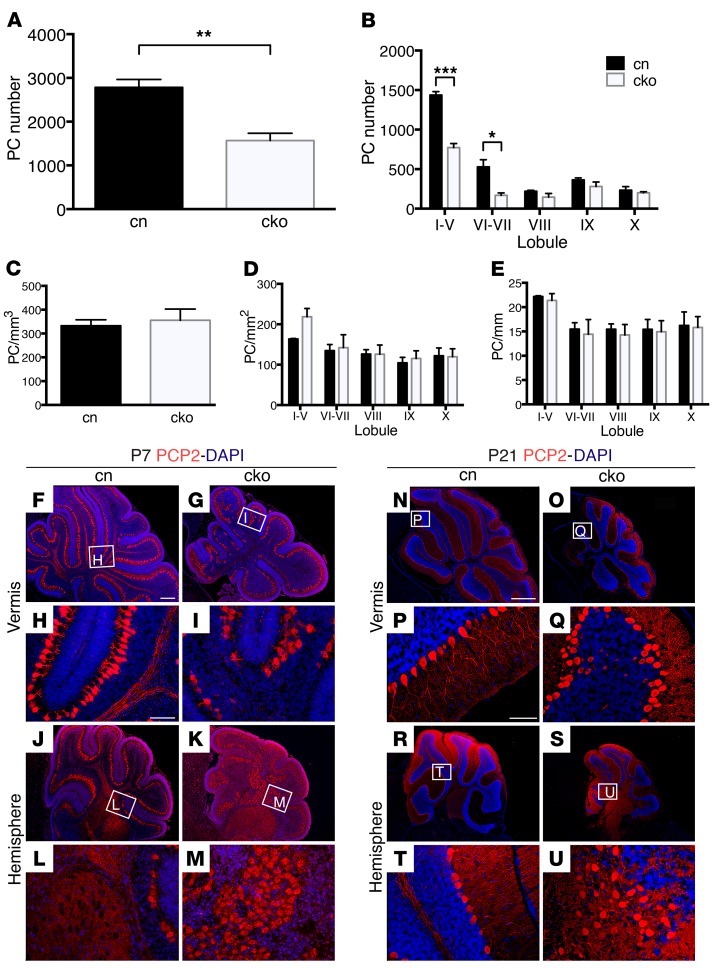
Deletion of *Chd7* from GCps results in PC abnormalities. (**A**) PC counts in cn and cko mouse P21 cerebella. Note the significant reduction in total PC number in the cko. (**B**) Lobule-specific PC counts. Note the significant reduction in PC number in the anterior and central lobules of the cko. (**C**–**E**) PC density in relation to the total vermis volume (mm^3^) (**C**), lobule granule cell area (mm^2^) (**D**), and length of the PC layer (mm) (**E**) (*n* ≥ 5 per genotype). *P < 0.05, **P < 0.01, ***P < 0.001, Student’s *t* test. (**F**–**U**) Examples of Purkinje cell protein 2 (PCP2) immunostains to visualize PCs in P7 cerebellar sections through the vermis (**F**–**I**) or hemispheres (**J**–**M**), with nuclear DAPI counterstain, anterior to the left. Magnified views of PC layers indicated by white boxed areas in corresponding low-power images are depicted in **H**, **I**, **L**, and **M**. Note the occasional disorganized PC distribution in the cko vermis (**I**), and more pronounced clusters of mislocalized PCs in the cko cerebellar hemispheres (**M**). (**N**–**U**) Examples of PCP2 immunostains to visualize PCs in P21 cerebellar sections through the vermis (**N**–**Q**) or hemispheres (**R**–**U**), with nuclear DAPI counterstain, anterior to the left. Magnified views of PC layers indicated by white boxed areas in corresponding low-power images are depicted in **P**, **Q**, **T**, and **U**. Note the occasional multilayered PC distribution in the cko vermis (**Q**), and more pronounced clusters of mislocalized PCs in the cko cerebellar hemispheres (**U**). Scale bars: 300 μm (**F**), 100 μm (**H** and **P**), 1 mm (**N**).

To determine the impact of the diminished production of granule neurons in the cerebellum on PCs, PC number and distribution were examined. Total PC numbers were reduced in cko cerebella at P21 (Figure 3A). A finer analysis of PC numbers across different cerebellar regions revealed that the reduction in PC number was due to fewer PCs in lobules I–VII of cko animals (Figure 3B), in agreement with the pronounced hypoplasia of anterior and central cerebellar lobules in the mutants (Figure 2D). PC density was not altered, indicating that the reduction in PC numbers remained proportional to the reduced cerebellar size (Figure 3, C–E). PCs in the cko cerebellar vermis were organized in monolayers for most of the cerebellum, with small regions of slightly disorganized cells observed at P7 (Figure 3, F–I) and P21 (Figure 3, N–Q). By contrast, large patches of disorganized PCs were present in the hemispheres at P7 (Figure 3, J–M) and P21 (Figure 3, R–U).

To understand the cause of these abnormalities, we investigated the distribution of LHX1/5^+^ PC progenitors over time as they migrate from their site of origin, the VZ toward the pial surface (23). This analysis revealed a relatively normal PC distribution, including formation of the PC plate under the pial surface at E16.5 (Supplemental Figure 7, A–H). Abnormal PC organization was evident by E18.5, with more dispersed PC progenitors in the central vermis (Supplemental Figure 7Q) and apparently mislocalized progenitors in the hemispheres (Supplemental Figure 7, S and T), consistent with the altered PC distributions seen in these regions at later stages. As CHD7 is also expressed in cells in the VZ, we asked whether nestin-*Cre* cko mice, in which *Chd7* has been deleted from these progenitors in addition to GCps, exhibited more pronounced PC developmental defects. Indeed, although a clear PC plate still formed in the medial cerebellum by E16.5 (Supplemental Figure 7I), PC distribution was clearly irregular in the rest of the cerebellum (Supplemental Figure 7, J–L). Mislocalized cells were evident in the vermis and hemispheres by E18.5 (Supplemental Figure 7, U–X). Together, these findings suggested that the deletion of *Chd7* from the early VZ progenitors contributed to PC defects in nestin-*Cre* cko mice, although we cannot rule out the possibility that the deletion of *Chd7* from the rhombic lip stream and EGL earlier than in *Math1-Cre* cko mutants also contributed to these defects.

### GCp-specific Chd7 conditional mouse mutants exhibit motor delay and coordination deficits.

**Figure 4:**
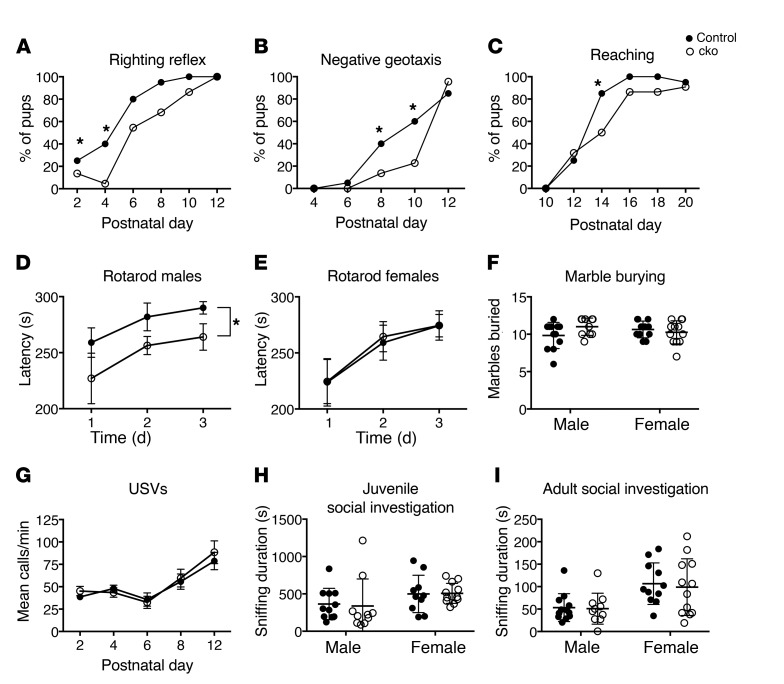
Deletion of *Chd7* from GCps leads to developmental delay and motor coordination deficits. Behavioral assessments of separate cohorts of adult (**D**–**F**, **H**, and **I**) cn (male *n* = 12, female *n* = 11) and cko (male *n* = 10, female *n* = 12) mice and pups for analysis of developmental milestones (cn *n* = 20, cko *n* = 22) or ultrasonic vocalizations (USVs) (cn *n* = 21, cko *n* = 23). (**A**–**C**) The percentage of pups at the indicated postnatal ages demonstrating the full righting reflex (**A**), a score of 1 or higher for negative geotaxis (**B**), or the full reaching response (**C**). Note the delay in acquiring these motor milestones in cko compared with cn animals (*P ≤ 0.05, χ^2^ tests). (**D** and **E**) The mean latency of male (**D**) and female (**E**) mice to fall from the rotarod. Note the significant difference between the male cko and cn animals (*P ≤ 0.05, repeated-measures ANOVA, with Student’s *t* test as post hoc analysis). (**F**) The mean number of ultrasonic vocalizations per minute at the indicated postnatal ages for cn and cko mice (repeated-measures ANOVA). (**G**) Average number of marbles buried within 30 minutes. Note no significant difference between the groups (between-subjects ANOVA). (**H** and **I**) The mean duration of total social investigation of an age- and sex-matched novel conspecific at P21 (**H**) and in adulthood (**I**). No significant difference between the cn and the cko groups was observed (repeated-measures ANOVA).

Next, we asked whether the cerebellar defects were sufficient to cause behavioral abnormalities in these mice. Mutant mouse pups exhibited normal growth (Supplemental Figure 8A). Pups were assessed for the acquisition of developmental milestones, which revealed a delay in acquiring the righting reflex (Figure 4A), the ability to turn around when placed head facing downward on a sloping platform (negative geotaxis; Figure 4B), and the ability to reach out toward an object (Figure 4C). To determine whether adult cko animals had any motor deficits, their motor coordination on a revolving rotarod was examined. Male cko mice performed significantly worse on this test than their sex-matched control littermates (Figure 4D), whereas female cko mice showed no difference compared with controls (Figure 4E). There were no significant differences in body weight or grip strength between control and cko animals (Supplemental Figure 8, B and C), excluding general muscle weakness as a potential cause of this motor phenotype. The performance of cko animals of both sexes improved to a similar extent over the 3 days of testing, indicating that these mutants had no deficit in motor learning. Mutant animals showed no signs of repetitive behaviors (Figure 4F) or anxiety (Supplemental Figure 8, D and E).

Cerebellar defects and dysfunction have also been proposed to be associated with autism spectrum disorders (reviewed in ref. 32). We therefore also performed tests to assess social interactions and communication. Mice communicate via vocalizations in the ultrasonic and sonic ranges, particularly in social situations such as when pups are separated from the mother (33). Mutant pups exhibited no difference in ultrasonic vocalizations (Figure 4G). Social investigation of conspecific age- and sex-matched mice by juvenile cko mice was normal (Figure 4H), as were reciprocal social investigations (Figure 4I) and sociability in the 3-chamber social approach test (Supplemental Figure 8F) in adult mice. In contrast to the *Chd7* heterozygous mouse models (34, 35), no differences were observed in olfactory discrimination tests (Supplemental Figure 8, G and H), consistent with the cerebellar-specific *Chd7* deletion in these mutants.

In addition to the well-established role of the cerebellum in sensorimotor function, structural and functional studies have also implicated the cerebellum in cognition and spatial processing (36). In humans, cognitive tasks tend to activate central cerebellar lobules VI, VII, and Crus I/II (reviewed in refs. 37, 38). Given the particularly strong phenotypes seen in the central cerebellar lobules of the cko animals, we also evaluated the performance of these mutants in the Morris water maze test, a cognitive task that assesses visual-spatial processing and memory. These conditional mutants exhibited no deficits in this task compared with control animals (Supplemental Figure 8I).

In conclusion, our behavioral tests indicate that cerebellar hypoplasia and PC hypocellularity in *Chd7* cko mutants are associated with developmental delay and motor coordination. By contrast, these defects are not sufficient to cause deficits in social behaviors or cognition.

### CHD7 controls gene expression levels in GCps.

**Figure 5:**
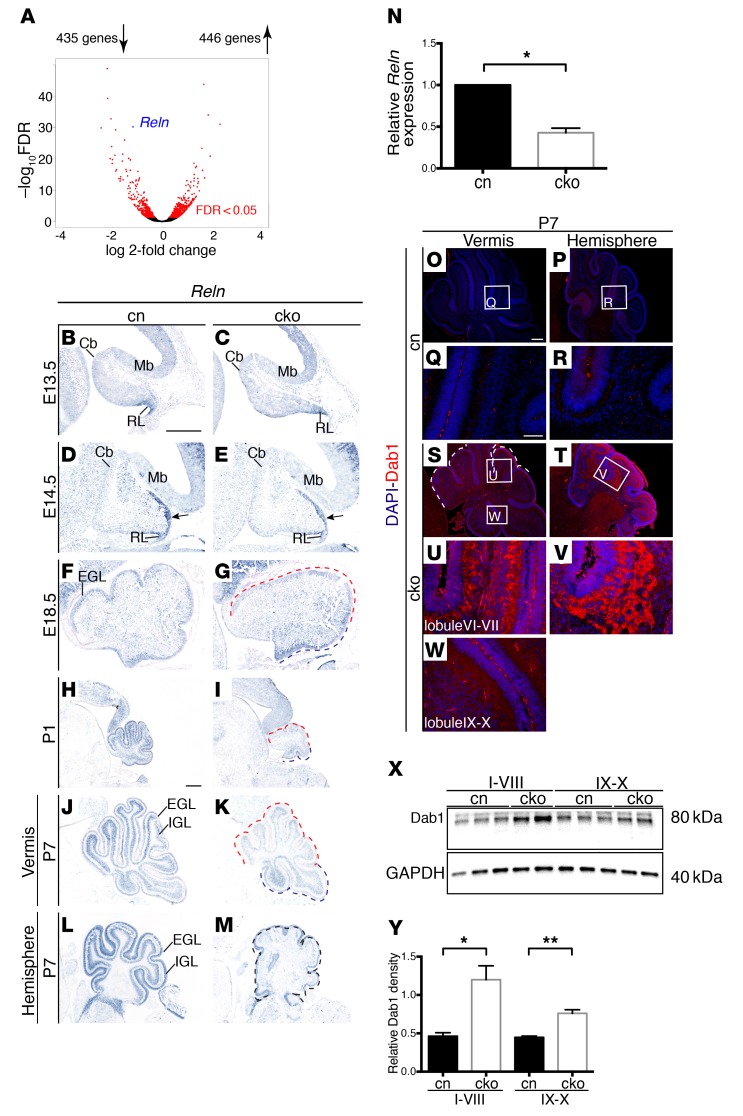
CHD7 regulates *Reln* gene expression in GCps. (**A**) Volcano plot of RNA-Seq data comparing gene expression of purified GCps from P7 cko and cn mice. Red points indicate genes with statistically significant (FDR < 0.05, *n* = 2) differences in expression. *Reln* is highlighted in blue. (**B**–**M**) In situ hybridization with an antisense *Reln* probe on sagittal cerebellar sections at the indicated time points. Note reduced *Reln* expression in the rhombic lip stream at E14.5 (arrow in **E**), anterior vermis of E18.5, P1, and P7 cerebella (red dashed line in **G**, **I**, and **K**), and all lobules of the hemispheres at P7 (**M**), with normal expression in the posterior vermis (navy dashed line in **G**, **I**, and **K**). IGL, internal granule layer. (**N**) Quantification of *Reln* transcript levels in purified P7 GCps confirms significant reduction in *Reln* expression in cko GCps (3 samples of purified GCps extracted from pooled cerebellar samples of each genotype). (**O**–**W**) Immunostaining for DAB-1 on sagittal sections of P7 mouse cerebella. High-magnification views of indicated regions in **O**, **P**, **S**, and **T** are shown in **Q**, **R**, and **U**–**W**, respectively. Increased DAB-1 protein levels in the anterior vermis lobules and hemisphere of cko mice are indicative of reduced RELN signaling. (**X**) Western blot analysis of DAB-1 protein levels in lobules I–VIII and IX–X. Note the increase in protein levels in cko samples (*n* = 3) compared with cn (*n* = 2), with more marked increases identified in lobules I–VIII of the cko. (**Y**) DAB-1 protein levels quantified relative to GAPDH. *P < 0.05, **P < 0.01, Student’s *t* test. Scale bars: 300 μm (**B**, **H**, and **O**), 100 μm (**Q**).

*Chd7* encodes a chromatin remodeling factor that has been postulated to function as a “transcriptional rheostat” to maintain developmental gene expression at physiological levels (9). Thus, to identify CHD7-regulated genes in GCps, a direct comparison of the *Chd7*-deficient and control transcriptome was performed by RNA sequencing (RNA-Seq). RNA was isolated from purified GCps at P7, when sufficient numbers of pure, primary GCps can be isolated ex vivo. A total of 881 coding transcripts with significantly changed expression (FDR < 0.05) were identified in *Chd7*-deficient GCps, compared with controls. Nearly equal numbers of genes were downregulated (*n* = 435) or upregulated (*n* = 446) (Figure 5A and Supplemental Table 2).

This experiment revealed significant downregulation of the *Reln* gene (labeled in blue in Figure 5A). Homozygous mutation of the *RELN* gene is associated with pronounced cerebellar hypoplasia in humans and mice (19, 39), suggesting that *Reln* downregulation might contribute to cerebellar hypoplasia in *Chd7* cko mutants. In situ hybridization studies revealed a subtle reduction of *Reln* expression in the EGL already at E14.5 (Figure 5, D and E), E18.5 (Figure 5, F and G), and substantial downregulation in *Chd7*-deficient GCps in the anterior and central cerebellum at P1 (Figure 5, H and I) and P7 (Figure 5, J and K). In agreement with the efficient deletion of *Chd7* throughout the lateral cerebellum (Supplemental Figure 3J), *Reln* expression was strongly downregulated in cerebellar hemispheres (Figure 5, L and M). Quantitative RT-PCR analysis showed that *Reln* expression was reduced by over 50% in cko GCps, compared with control GCps (Figure 5N). RELN signaling is associated with the degradation of the intracellular protein DAB-1 (21, 40–42), and therefore, reduced RELN signaling should result in increased levels of DAB-1 protein. Immunostaining with a DAB-1–specific antibody revealed a clear upregulation of DAB-1 protein in PCs in the anterior and central vermis lobules and hemispheres of *Chd7* cko cerebella (Figure 5, S–V), compared with lobule X (Figure 5W) and control cerebella (Figure 5, O–R). These findings were corroborated by immunoblotting, which also revealed increased DAB-1 protein in the cko cerebellum, compared with the control cerebellum (Figure 5, X and Y). These observations confirmed that *Reln* downregulation was sufficient to cause a substantial reduction in RELN signaling in the developing cerebellum and identify CHD7 as an important regulator of *Reln* gene expression and signaling levels in the developing cerebellum.

### Reduced RELN signaling contributes to cerebellar hypoplasia in Chd7 cko mice.

**Figure 6:**
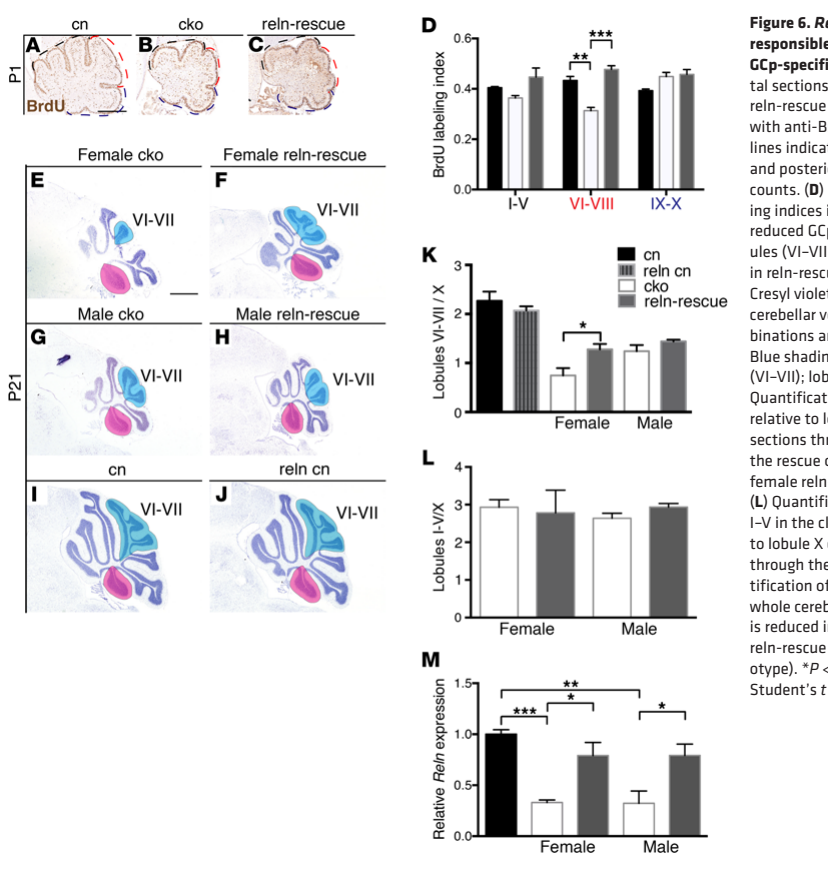
*Reln* downregulation is partly responsible for cerebellar hypoplasia in GCp-specific *Chd7* cko mice. (**A**–**C**) Sagittal sections of BrdU-treated cn, cko, and reln-rescue cerebella at P1, immunostained with anti-BrdU antibody (brown). Dashed lines indicate anterior (black), central (red), and posterior (navy) cerebellum used for BrdU counts. (**D**) Quantification of the BrdU labeling indices in regions indicated in **A**–**C**. Note reduced GCp proliferation in the central lobules (VI–VIII) of the cko mice and restoration in reln-rescue mice (*n* = 3 per genotype). (**E**–**J**) Cresyl violet–stained sagittal sections of the cerebellar vermis at P21; sex-genotype combinations are indicated, anterior to the left. Blue shading indicates the central lobules (VI–VII); lobule X is highlighted in pink. (**K**) Quantification of the volume of lobules VI–VII relative to lobule X calculated from serial sections through the cerebellar vermis. Note the rescue of the central lobule hypoplasia in female reln-rescue mice (*n* = 3 per genotype). (**L**) Quantification of the volume of lobules I–V in the cko and reln-rescue mice relative to lobule X calculated from serial sections through the cerebellar vermis. (**M**) Quantification of *Reln* transcript levels in E18.5 whole cerebella. Note that *Reln* expression is reduced in the cko and restored in the reln-rescue irrespective of sex (*n* = 5 per genotype). *P < 0.05, **P < 0.01, ***P < 0.001, Student’s *t* test.

To test whether reduced RELN signaling was responsible for cerebellar hypoplasia in *Chd7* cko mice, we sought to rescue this phenotype by increasing *Reln* expression in these mutants in vivo. For this purpose, we used a nestin-*Reln* transgenic mouse (*RelnTG*) reported to misexpress *Reln* throughout the developing cerebellum at 10%–20% of WT levels until P5 (43). Despite its low, transient, and ectopic expression, this transgene was capable of partially rescuing the cerebellar hypoplasia in homozygous reeler mice (43). We therefore generated *Math1-Cre Chd7^fl/fl^ RelnTG* (referred to as reln-rescue) mice. We first asked whether the transgene could normalize the GCp proliferation defect observed in *Chd7* cko mutants at P1 (Figure 6, A–D). The defect was fully corrected in reln-rescue mice (Figure 6D). To examine whether transient transgenic *Reln* expression had any lasting effect on the size of these central cerebellar lobules, P21 cerebella were examined. As *Chd7* is not deleted (Supplemental Figure 3, H and R) and *Reln* expression is not altered (Figure 5K) in lobule X as a result of the inefficiency of the *Math1-Cre* transgene in the posterior cerebellum, we measured the size of the central lobules as a fraction of lobule X as internal control. This analysis revealed a significant increase in the relative size of the central lobules VI–VII in female reln-rescue mice, compared with *Chd7* cko mice (Figure 6, E, F, and K). By contrast, *RelnTG* expression did not significantly increase the size of the central lobules in male mice (Figure 6, G, H, and K). Control *RelnTG* mice had a normal cerebellar size and morphology (Figure 6, I–K). The relative size of anterior lobules (I–V) was not rescued by *RelnTG* expression (Figure 6L), consistent with the lack of significant effect on GCp proliferation here (Figure 6D). Quantification of *Reln* transcript levels in E18.5 cerebella confirmed an almost 2-fold upregulation of *Reln* transcripts in *RelnTG* mice compared with mutants (Figure 6M), consistent with the near-complete rescue of proliferation at P1 (Figure 6D). Importantly, there was no difference in *Reln* levels between male and female cko cerebella, indicating that sex differences in *Reln* expression levels could not explain the sex-specific rescue effects seen (Figure 6M). In summary, our genetic studies confirmed that reduced *Reln* expression significantly contributes to the proliferation deficit of *Chd7*-deficient GCps in central cerebellar lobules and may affect cerebellar growth in a sex-specific manner.

### CHD7 is required for the maintenance of DNA accessibility at the Reln promoter.

CHD7 is thought to function primarily by modulating DNA accessibility at developmental regulatory elements through its ATP-dependent chromatin remodeling activity. We hypothesized that CHD7 assists in the maintenance of an “open,” accessible chromatin structure at CHD7-regulated regulatory elements in primary GCps. To address this question, DNA accessibility in control and *Chd7* cko GCps was compared using an assay for transposase-accessible chromatin with high-throughput sequencing (ATAC-Seq) (44). In total, 142,047 ATAC-Seq peaks, i.e., regions where the DNA is accessible to transposase, were identified in the control cells (Supplemental Table 3). We compared the ability of ATAC-Seq to identify regions of accessible DNA with DNase I hypersensitive sites sequencing (DNase-Seq) data recently published from GCps (45). Our analysis of DNase-Seq data identified 50,022 accessible peaks in P7 GCps, 88% (43,782) of which were within 100 bp of an ATAC-Seq peak, indicating that ATAC-Seq can reliably identify accessible regions.

**Figure 7:**
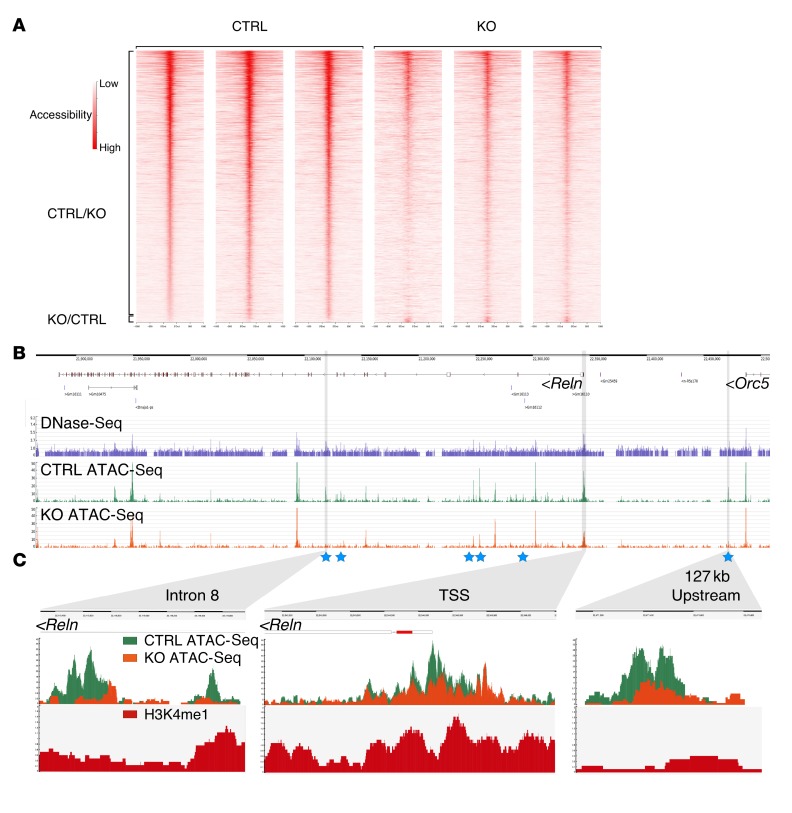
CHD7 deletion alters chromatin organization and reduces DNA accessibility at the *Reln* locus. (**A**) Heatmaps representing all significantly changed ATAC-Seq peaks ± 500 bp between control (CTRL) and cko (KO) GCps (*n* = 3). Note reduced DNA accessibility in KO samples for the majority of peaks (CTRL/KO peaks), with a few showing increased DNA accessibility in the KO (KO/CTRL peaks). (**B**) DNase-Seq (purple) (45) and ATAC-Seq reads mapped over the *Reln* gene in control (CTRL ATAC-Seq, green) and cko (KO ATAC-Seq, orange) P7 GCps. Peaks are indicative of regions of “open” chromatin with high DNA accessibility. Blue stars indicate ATAC-Seq peaks that are significantly different in CTRL and KO cells. (**C**) Visualization of normalized CTRL (green) and KO ATAC-Seq (orange) reads, and H3K4me1 ChIP-Seq reads from WT GCps (red) that mapped to intron 8, the *Reln* transcriptional start site (TSS), and a region 127 kb upstream of the *Reln* TSS. Note significantly reduced DNA accessibility at the intronic and upstream regions in KO cells compared with CTRL cells.

Having validated the ATAC-Seq approach, we asked whether DNA accessibility was significantly altered at any of these regions in *Chd7*-deficient GCps. We identified 4,921 regions that showed significantly reduced DNA accessibility in *Chd7*-deficient GCps (CTRL/KO, Figure 7A), and only 210 regions with increased accessibility (KO/CTRL, Figure 7A). This finding suggested that CHD7 primarily functions in the maintenance of open, accessible chromatin states in the genome.

Next, we determined whether any of these differentially regulated regions were in close vicinity of differentially expressed genes. Four hundred seventy-two (9.6%) of the peaks with reduced accessibility mapped to a location within 1 kb of a differentially expressed gene identified by RNA-Seq. Of these regions, 333 (70.6%) and 139 (29.4%) were located within 1 kb of a downregulated and upregulated gene, respectively. Thus, although there appears to be some association of downregulated gene expression with reduced DNA accessibility, this is by no means absolute. A similar observation was made for regions that showed increased accessibility in cko cells. Of the 210 peaks, 20 (9.5%) mapped to a location within 1 kb of a differentially expressed gene. Of these peaks, 11 (55%) and 9 (45%) were within 1 kb of a downregulated and upregulated gene, respectively.

Finally, we asked whether the accessibility of any potential regulatory elements at the *Reln* locus was altered in *Chd7*-deficient GCps. A direct comparison of DNase-Seq and ATAC-Seq profiles over the *Reln* locus (Figure 7B) clearly showed the superiority of the ATAC-Seq approach in being able to unambiguously identify regions of DNA accessibility. A statistically significant reduction in DNA accessibility was evident at 6 putative regulatory elements at the *Reln* locus (blue stars, Figure 7B). As examples of regions with reduced DNA accessibility, zoomed-in views of a region in intron 8 and 127 kb upstream of the transcriptional start site are shown (Figure 7C). Although accessibility at the *Reln* transcriptional start site also appears to be reduced, these changes did not reach statistical significance. Finally, we confirmed that these regions were positive for the H3K4me1 histone modification, a marker of regulatory regions to which CHD7 can be recruited (8). Thus, our findings are consistent with a model whereby CHD7 is recruited to regulatory elements at the *Reln* locus, where it directly modulates chromatin structure. Together, these observations suggest a crucial role for CHD7 in maintaining an accessible, “open” chromatin structure throughout the genome and at the *Reln* locus.

## Discussion

In this article we define an important role for CHD7 in the expansion of the GCp pool in the perinatal cerebellum. Together with our previous work (11), we conclude that CHD7 controls cerebellar growth at 2 distinct stages of cerebellar development. CHD7 is essential for the maintenance of rhombomere 1 identity and *Fgf8* expression in the embryonic isthmus organizer, with disruption of the latter predisposing the embryo to cerebellar vermis hypoplasia. In this article, we show that CHD7 expression becomes localized to cerebellar GCps where it regulates GCp proliferation and survival. CHD7 deletion from GCps as well as PC progenitors in the VZ from E12.5 with nestin-*Cre* resulted in a more severe phenotype, potentially implicating CHD7 expression in the VZ in cerebellar development. *Chd7* gene deletion specifically in the cerebellar VZ using *Ptf1a-Cre* will be necessary to address this possibility directly. We show that reduced RELN signaling in CHD7-deficient GCps can partially account for the cerebellar hypoplasia. The *RelnTG* line we used in these experiments does not express the *Reln* transgene past P5, likely explaining the limited phenotypic rescue achieved by P21. Nevertheless, the possibility that other genes deregulated in CHD7-deficient GCps also contribute to reduced GCp expansion remains likely.

The exact mechanism whereby reduced RELN signaling results in GCp proliferation defects is not known. In homozygous reeler (*Reln^rl/rl^*) mutants, PC migration from the VZ is disrupted, presumably leading to a deficit in PC-derived mitogenic signals (23). PC positioning is abnormal in our mutants, especially in the cerebellar hemispheres, suggesting that PC disruption may contribute to the hypoplastic phenotype. Expression analysis of several genes transcriptionally regulated by SHH signaling (*Gli1*, *Ptch1*, and *Mycn*) by in situ hybridization and quantitative RT-PCR revealed no significant downregulation in their expression, suggesting that PC disruption in our mutants may affect GCp proliferation in an SHH-independent manner. Alternatively, PC defects may not be responsible for GCp proliferation defects, and RELN downregulation may affect GCp proliferation in a cell-autonomous manner. Consistent with this latter possibility, transcripts encoding the RELN receptors ApoER2, VLDLR, and ephrin B1, B2, and B3 are present in GCps (RNA-Seq data, Supplemental Table 2). GCp-specific deletion of *Reln* and combinations of genes encoding the different RELN receptor genes will be necessary to examine this possibility.

The finding that increased *Reln* expression had a significant effect on the size of the central lobules in female cko mice, but not males (Figure 6K), suggested that perhaps female mice were more sensitive to changes in *Reln* expression. Interestingly, sex-genotype interactions appear to be important in the expression of psychiatric diseases associated with *RELN*. Specifically, genome-wide association studies have identified common variants in the *RELN* gene that increase the risk of schizophrenia and bipolar disorder only in women (46, 47). Sex-specific differences in the anatomical and behavioral phenotype of heterozygous reeler mice (*Reln^rl/+^*) have also been reported (48). PC degeneration has been identified from postnatal day 15 in male *Reln^rl/+^* mice, and motor dysfunction has been reported in adult males (49, 50). Reduced *Reln* expression and neuroactive steroid levels appear to be critical in the pathophysiology of the *Reln^rl/+^* phenotype, since administration of 17β-estradiol protects against PC degeneration in males (49). Taken together, these studies are indicative of a complex interplay between RELN signaling and sex hormones during cerebellar development. In the present study, we ruled out a simple model whereby *Reln* gene expression in GCps differs in the 2 sexes (Figure 6M).

We report here that adult GCp-specific *Chd7* cko mutant mice with cerebellar hypoplasia exhibit a range of behavioral deficits. The identification of developmental motor delay and mild motor deficits in these animals implies that cerebellar hypoplasia may underlie some of these features in CHARGE syndrome patients. We propose that clinical studies to assess and compare the severity of motor delay and coordination deficits in CHARGE syndrome patients with or without cerebellar hypoplasia will be important to delineate the contribution of cerebellar defects to these deficits. Our findings may also have implications for understanding other clinical features of CHARGE syndrome. We note that several CHD7-regulated genes implicated in other cell types and tissues affected in CHARGE syndrome (e.g., *Sema3a*, neural crest; ref. 51) are also dysregulated in GCps, perhaps indicative of common gene regulatory mechanisms in different developmental contexts.

Interestingly, we detected no deficits in social behavior in these mutants. This finding is important to consider in the context of cerebellar anomalies described in patients diagnosed with autism. For example, hypoplasia of the central vermis lobules VI–VII has been reported in autism spectrum disorder (ASD) cases (52, 53). Our data suggest that marked hypoplasia of the central cerebellar lobules, associated with reduced PC numbers in these lobules, is not sufficient to cause social deficits in mice. This conclusion has to be tempered by consideration of several caveats. First, mouse models might not be ideal for addressing this question, as the nature of cerebellar–prefrontal cortex connections may be qualitatively or quantitatively different from that in primates and humans (12). Second, the behavioral tests used to evaluate social investigations in mice may not be sensitive enough to detect appropriate changes in behavior. Another neuroanatomical anomaly frequently reported in the context of ASD is the reduction in PC number in postmortem brain samples from ASD patients (reviewed in ref. 54). It was reported recently that conditional deletion of the *Tsc1* gene from cerebellar PCs alone was sufficient to cause autism-like behaviors in a mouse model (55). Our data indicate that a reduction in PC number as a consequence of disruption of the normal developmental process is, by itself, not sufficient to cause autism-like social behaviors in mice.

A number of nucleosome remodeling factors appear to be critical for normal brain development, and mutations that disrupt their normal function are associated with neurodevelopmental disorders (56). Identifying the changes in chromatin structure that result from disrupted function of these factors is an important step toward understanding the mechanisms that underlie these conditions. The prevailing model for CHD7 function is that CHD7 remodels chromatin by sliding nucleosomes along chromatin to modulate DNA accessibility at key developmental regulatory elements (57). We identified a striking reduction in DNA accessibility throughout the genome in CHD7-deficient cells, consistent with a role for CHD7 in the maintenance of regions of open, accessible chromatin. This finding is in keeping with the activity of the CHD7 homolog, kismet, that was operationally identified as a Trithorax family member that functions as an antagonist of Polycomb factors (58), which promote chromatin compaction and gene repression (59).

In conclusion, we have defined a critical role for CHD7 in cerebellar GCp expansion and identified CHD7 as a regulator of *Reln* gene expression, thereby linking CHD7 to a pathway associated with cerebellar hypoplasia and neuropsychiatric disease in humans. Behavioral analyses of GCp-specific mouse mutants revealed important impacts of cerebellar hypoplasia on motor development and function. These findings have implications for understanding the neurodevelopmental basis of motor delay in CHARGE syndrome. Intriguingly, our data do not support a view where cerebellar hypoplasia per se is sufficient to cause behavioral deficits in the social domain, at least not in mouse models. Finally, we identify a crucial molecular function of CHD7 in the maintenance of open, accessible chromatin in primary neuronal progenitors.

## Methods

### Mice.

The *Chd7^flox^* (28), nestin-*Reln* transgenic (*RelnTG*) (43), *Math1-Cre* (30), and nestin-*Cre* (29) mouse lines, provided by Pete Scambler (Institute of Child Health, London, United Kingdom), Tom Curran (Children’s Hospital of Philadelphia, Philadelphia, Pennsylvania, USA), David Rowitch (UCSF, San Francisco, California, USA), and Gill Bates (University College London, London, United Kingdom), respectively, were genotyped by PCR using tail or ear DNA. Mouse lines were backcrossed with C57BL/6J mice for at least 3 generations. Conditional neural-specific mutants were obtained from nestin-*Cre Chd7^fl/+^* × *Chd7^fl/fl^* matings. Conditional GCp-specific mutants were obtained from *Math1-Cre Chd7^fl/fl^* × *Chd7^fl/fl^* matings. For behavioral tests, sex-balanced matings were used to produce animals for testing to control for any sex-specific parental genotype effects. Juvenile (3-week-old) C57BL/6J mice used as conspecifics were purchased from Charles River Laboratories and group-housed by sex.

Mice were bred in the Biological Services Unit at Guy’s Campus or the Institute of Psychiatry, Psychology & Neuroscience, King’s College London.

### Histology.

Samples were dissected in PBS, fixed overnight in 4% paraformaldehyde (PFA) at 4°C, dehydrated, and embedded in paraffin wax. Serial, sagittal sections were cut at 10 μm and left to dry overnight at 42°C.

### Volumetric analysis and PC counts.

The surface area of serial, Cresyl violet (0.1%)–stained sections was measured in ImageJ (NIH) and the vermis volume calculated by multiplication of the total surface area by the section thickness (10 μm). PCs were counted in 5 separate, nonadjacent 10-μm vermis sections (3 cerebella per genotype) in H&E-stained sections.

### Immunohistochemistry.

Sections were deparaffinized in xylene and rehydrated, heated in a sodium citrate buffer (pH 6.0), permeabilized in 0.2% Triton X in PBS (PBSTx), and blocked in 10% heat-inactivated goat serum (GS) in PBSTx for 1 hour before incubation in primary antibody in 5% GS-PBSTx overnight at 4°C. Antibodies were: rabbit anti–DAB-1 (1:150; Sigma-Aldrich, D1569), rabbit anti–Purkinje cell protein 2 (anti-PCP2) (1:200; gift from Brad Denker, Harvard University, Boston, Massachusetts, USA), mouse anti-BrdU (1:100; BD Biosciences 347580), and cleaved caspase-3 (Asp 175) (1:150; Cell Signaling Technology 9661). For DAB-1 and PCP2 detection, sections were incubated with Alexa Fluor–labeled secondary antibodies (1:200; Life Technologies) and counterstained with DAPI. Slides were mounted with Citifluor (Citifluor Ltd.), and fluorescent images were captured using a Nikon Eclipse 80i microscope with a Nikon Y-QT Hamamatsu C4742-95 camera. For BrdU and cleaved caspase antibodies, sections were incubated with polyclonal goat anti-mouse or goat anti-rabbit biotinylated secondary antibody (1:200; Dako) for 1 hour. Signal was amplified using the VECTASTAIN Avidin/Biotin Complex (ABC) kit (1:200; Vector Laboratories Ltd.), and visualized with 3,3′-diaminobenzidine substrate (0.03%; Sigma-Aldrich). Sections were then counterstained with hematoxylin (30 seconds), dehydrated, mounted in DPX, and imaged with a Nikon Eclipse 80i.

### BrdU.

Pregnant females or pups were given 1 i.p. injection of 50 mg/kg or 20 mg/kg 5′-bromo-2′-deoxyuridine (BrdU), respectively. Mice were sacrificed 1 hour later, and brains were fixed with 4% PFA overnight at 4°C, washed in PBS, and processed as described above. The BrdU labeling index was calculated by counting of the number of BrdU-labeled cells and the total number of cells in the EGL in 100-by-100-μm areas (2–3 fields per lobule) using a Nikon Eclipse 80i microscope. The BrdU-positive GCps were then plotted as a fraction of the total number of GCps per lobule.

### Western blot.

P7 cerebella were dissected in PBS and lobules IX and X separated from lobules I–VIII. Tissue was lysed in 8 M urea, 1% CHAPS, 50 mM Tris (pH 7.9) followed by the removal of DNA by centrifugation. Proteins (10 μg per lane) were resolved on a Mini-PROTEAN precast gel (Bio-Rad, United Kingdom) and transferred to a nitrocellulose membrane (Bio-Rad, United Kingdom). After blocking with 0.5% nonfat milk powder in TBS with 0.5% Tween-20 (TBST), the membrane was incubated with primary antibodies (rabbit anti–DAB-1 [C-terminal], Sigma-Aldrich, D1569, 1:5,000; rabbit anti-GAPDH, Abcam, ab181602, 1:10,000) in 5% BSA, TBST overnight at 4°C. After washing and incubation with HRP-conjugated secondary antibody (Millipore) for 1 hour at room temperature, HRP conjugates were detected using Clarity Western ECL reagent (Bio-Rad). Gels were visualized on a Bio-Rad gel doc system and images analyzed with Image Lab (Bio-Rad) and prepared using Adobe Photoshop.

### In situ hybridization.

In situ hybridization was performed using standard methods (60). DNA templates were produced by PCR amplification from mouse genomic DNA. The PCR primers used were: *Chd7* (exon3) forward 5′-TTGGTAAAGATGACTTCCCTGGTG-3′, reverse 5′-GTTTTGGCGTGACAGTTTTTGC-3′; *Reln* (exon 65) forward 5′-CAACAGAAGACGAAGGTCGCTTAG-3′, reverse 5′-ACAAGTAGGTCAAAGTCCAGCAGC-3′. Digoxigenin-labeled antisense probes for *Chd7* and *Reln* were produced by in vitro transcription using a DIG RNA labeling kit with T7 RNA polymerase (Roche).

### GCp purification and quantitative PCR.

P7 cerebella were dissected in DPBS (Gibco), and lobules IX and X were removed. The cerebella of the individuals from each genotype were pooled. Cells were dissociated using Papain I (100 U; Worthington) followed by trituration. Granule cell precursors were isolated by Percoll fractionation with a 35% and 65% Percoll gradient (Sigma-Aldrich) as previously described (14).

Total RNA was extracted from the purified granule cells of 3 samples of each genotype using Trizol (Invitrogen). cDNA was synthesized from 200 ng of RNA using the nanoScript 2 Reverse Transcription kit (Primerdesign Ltd.) with random hexamer primers. Quantitative RT-PCR was performed on a Stratagene Mx3000P (Agilent Technologies) using the Precision qPCR master mix with SYBR Green (Primerdesign Ltd.). All reactions were run in triplicate, and first-strand DNA synthesis reactions without reverse transcriptase were used as controls. The quantification cycle (Cq) threshold values were normalized to the housekeeping gene *Gapdh* to calculate the ΔCq value and the ΔΔCq calculated relative to the control sample. The primer sequences were: *Chd7* forward 5′-TCACCAGCCTTGGGCACAACTC-3′, reverse 5′-TAGCTGAGCGTTCTGTGCGCTG-3′; *Reln* forward 5′-TTACTCGCACCTTGCTGAAAT-3′, reverse 5′-CAGTTGCTGGTAGGAGTCAAAG-3′; *Gapdh* forward 5′-AGGTCGGTGTGAACGGATTTG-3′, reverse 5′-TGTAGACCATGTAGTTGAGGTCA-3′.

### RNA-Seq.

GCps were purified from P7 control (*Chd7^fl/+^*) and cko (*Math1-Cre Chd7^fl/fl^*) mice as described above. Total RNA was extracted from freshly isolated cells (5 × 10^6^ per cerebellum) with an Agilent Absolutely RNA miniprep kit (Agilent Technologies). RNA quality and integrity were assessed on a 2100 Bioanalyzer and 2200 Tapestation using an R6K screenTape (Agilent Technologies). After quantification on Qubit using Quant-iT RNA assay kit (Life Technologies), 2 μg total RNA was used from each sample for library preparation using an Illumina TruSeq kit. Libraries were resolved by agarose gel electrophoresis, and 250- to 550-bp DNA fragments were isolated and gel-purified with a Qiagen DNA gel extraction kit (Qiagen). Two libraries with unique barcodes were combined and sequenced per lane on a HiSeq2000 (Illumina) at the Biomedical Research Centre, King’s Health Partners (London, United Kingdom). RNA-Seq reads were aligned to the mouse reference genome (mm10) using TopHat (version 2.0.14) (61) with an expected inner distance between mate pairs of 150 bp and SD for the distribution on inner distances between mate pairs of 50 bp. The transcriptome index was created using Ensembl build 75. HTseq (version 0.6.1p1) (62) was used to estimate the number of reads (ftp://ftp.ensembl.org/pub/release-75/gtf/mus_musculus/). An intersection-strict mode was used and all reads with an alignment quality lower than 3 skipped. Differential expression was analyzed with DESeq2 1.8.1. (https://bioconductor.org/packages/release/bioc/html/DESeq2.html). Genes with an FDR less than 0.05 were considered differentially expressed. RNA-Seq, ChIP-Seq, and ATAC-Seq data have been deposited in the NCBI’s Gene Expression Omnibus (GEO 18162240).

### ChIP-Seq.

GCps were purified from P7 control (*Chd7^fl/+^*) mice as described above. Freshly isolated cells (2 × 10^7^ to 3 × 10^7^) of each genotype were fixed, chromatin isolated, and precipitated with an antibody against H3K4me1 (Abcam ab8895), and ChIP-Seq libraries were produced and sequenced as previously described (63). The ChIP DNA libraries were sequenced (50 bp, single-end) on an Illumina HiSeq 2000 sequencer at the New York University Genome Technology Center (New York, New York, USA). The reads were aligned to the mouse reference genome (mm10) using Bowtie (version 1.1.0), allowing only uniquely mapping reads, with an insert size of 1,000 bases. Default values were used for all other parameters. Following mapping, PCR duplicates were removed using the SAMtools “rmdup” command (64). Identification of enriched regions was performed by comparison of the ChIP to the input reads using MACS2 (2.1.0.20140616) (65), with a *P* value cutoff of 0.0001 and 3,90 as the model building parameters. The bedGraph output generated by MACS2 was used to visualize the ChIP and input signals.

### ATAC-Seq.

GCps were purified from P7 mice as described above. Intact nuclei were isolated (EZ Prep Nuclear Isolation Kit, Sigma-Aldrich) from 50,000 freshly isolated GCps of each genotype, and ATAC-Seq libraries were produced as previously described (44). Library quality was assessed on a 2100 Bioanalyzer and quantified using the KAPA library quantification kit (Kapa Biosystems). Six libraries were multiplexed (3 per genotype) and 75 bp paired-end reads were sequenced on a HiSeq4000 (Wellcome Trust Centre for Human Genetics, Oxford, United Kingdom). Reads were uniquely aligned to the mouse reference genome (mm10) using Bowtie (version 1.1.1) (66) with a maximum insert size for valid paired-end alignments of 2,000 and a maximum number of attempts of 200 to match an alignment for 1 mate with an alignment for the opposite mate. Peaks were called in 3 control replicates by comparing them to 3 cko samples and vice versa using MultiGPS (version 0.5) (67) with the minimum *q* value for reported peaks set at 0.05 and the mitochondrial genome excluded. The bedtools window function from BEDTools (version 2.17.0) (68) was used to identify ATAC-Seq peaks within 1,000 bp of differentially expressed genes (DEGs).

### DNase-Seq.

Raw reads were obtained from Frank et al. (45), and analyzed in the same way as the ATAC-Seq reads, except in the mapping step, where reads mapping with up to 4 locations were retained for further analysis.

### Heatmaps.

Reads that fell within ±500 bp of all changed ATAC-Seq peaks were plotted with ngs.plot.r (69), and regions were clustered according to the peak value of the first profile, using a global color scale across all plots.

### Behavioral tests.

Three batches of mice were used for testing of developmental milestones, recording of ultrasonic vocalizations, and testing of juvenile-adult behavior. For the juvenile-adult behavior, tests were carried out in the following order: juvenile social investigation, rotarod, open field, light/dark test, adult social investigation, 3-chamber social approach, marble burying, olfactory habituation/dishabituation, Morris water maze, and grip strength.

Using a battery of tests (70, 71), key behavioral milestones observed in mice prior to weaning (P2–P20) were used to assess core trajectories in the development of motor and sensory abilities (70) (see Supplemental Methods for detailed information). Ultrasonic vocalizations were recorded in pups across 3-minute sessions in response to social separation from the mother and siblings at P2, P4, P6, P8, and P12, in a dimly lit (<10 lux) soundproof chamber. The mean number of calls was used as a quantitative measure of communicative ability, as described previously (72). Motor coordination and learning were assessed on a rotating rod (Ugo Basile) as described previously (73) when the mice reached 42–45 days of age. The latency to fall for any particular day was calculated as the mean of 2 trials. Repetitive digging behavior to bury marbles was measured (74) in a dimly lit test room (<10 lux). Twelve blue glass marbles were arranged in a symmetrical 4-by-3-cm grid on top of 5-cm-deep sawdust (Litaspen premium, Datesand Ltd.) in a clean, standard housing cage (32 × 16 × 14 cm). Each mouse was given 30 minutes to freely explore the cage, and the number of marbles buried was counted. Social investigation was assessed at different ages to evaluate trajectories across developmental stages from juvenile to adulthood (33, 75, 76). Social investigation of age-matched C57BL/6J sex-matched conspecifics was assessed in juvenile test mice (P21) as described previously (75). Social investigation of juvenile C57BL/6J sex-matched conspecifics was assessed in adult test mice as described previously (76).

### Statistics.

Statistical tests used are reported in the figure legends, where all data presented indicate mean ± SEM, unless otherwise specified. A *P* value less than 0.05 was considered significant. All representative experiments were conducted on a minimum of 3 separate occasions with a minimum of 3 individual samples. All statistical analysis was conducted using SPSS (Statistics 22, IBM).

### Study approval.

All animal housing and experimental procedures were performed in compliance with the local ethical review panel of King’s College London, and the UK Home Office Animals Scientific Procedures Act 1986. The work was carried out under licenses (PPL70/6694 and PPL70/7184), and all efforts were made to minimize animal suffering and to reduce the number of animals used.

## Author contributions

DEW designed and performed most experiments, analyzed results, and prepared figures. KLHR and CF designed, performed, and analyzed behavioral experiments. BPS, KLHR, and MAB designed and performed ATAC-Seq experiments, and analyzed next-generation sequencing (NGS) data with C. Mohan, HH, and FCW. SK performed the analysis of the nestin-*Cre* cko mice. TY, AS, and MESS performed initial analysis of mutant phenotypes and contributed data to the manuscript. AC performed and analyzed ultrasonic vocalizations and contributed to developmental milestone experiments. C. Michetti, MS, and OK analyzed behavioral data. ACM analyzed and interpreted RNA-Seq data. WWT and DR assisted with ChIP-Seq experiments. MLS supervised ultrasonic vocalization analysis and assisted with behavioral data analysis. HV and IM contributed to data analysis. DEW, KLHR, C. Mohan, BPS, HH, HV, IM, and CF contributed to the writing of the manuscript. MAB designed the study, performed the RNA-Seq and ChIP-Seq experiments, supervised data analysis and interpretation, and wrote the manuscript. All authors read and approved the final version.

## Supplementary Material

Supplemental data

Supplemental Table 2

Supplemental Table 3
