# Supplementary material for: The chromatin remodeling factor CHD7 controls cerebellar development by regulating reelin expression
Source: J Clin Invest. 2017 Feb 6;127(3):874–87. doi: 10.1172/JCI83408 (PMC5330721; doi:10.1172/JCI83408)
Supplement: Supplemental data [file jci-127-83408-s001.pdf]

Supplemental information

Methods and Materials

Mice: Mice were housed in standard cages measuring 32×16×14cm with sawdust (Litaspen premium, Datesand Ltd, Manchester), a cardboard shelter and additional bedding material (Sizzlenest, Datesand Ltd, Manchester) with *ad libitum* access to water and food (Rat and Mouse No. 1 and 3 Maintenance Diet for test and breeding mice respectively, Special Diet Services, Essex, UK). The housing and test rooms were maintained at constant room temperature (21 °C) and humidity (45%) and kept under a regular light/dark schedule with lights on from 07:30 to 19:30 hours (light = 270 lux). The adult test batch mice were weaned at P21 and housed in same-sex groups of 2-3. Prior to the start of the test battery (at around 40 days of age), mice were singly-housed. Animals were singly housed when adult to avoid any potential confounds from social hierarchies, which could influence the controlled assessment of social behaviors (Brown, 1953). The estrous phase of the female mice was not checked in this study. However, it is unlikely that this affected results because there were no major effects in the variance between males and females. Sawdust was changed every other week but never on the day before, or the day of, testing and the enrichment (nesting material and house) was changed less regularly to minimize the disruption to the animals.

Behavior: Behavioral experiments were conducted between 08:30 and 18:30 in sound-proofed rooms under standard room lighting unless stated otherwise. Behaviors were recorded using a camera positioned above the test arenas and movement of each mouse tracked using EthoVision (Noldus Information Technologies bv, Wageningen, The Netherlands). After each individual test, boli and urine were removed from the test arena which was cleaned with 1% Anistel® solution ((high level surface disinfectant, Trisel Solution Ltd, Cambridgeshire, UK) to remove any odors. Experimenters were blind to the genotype of the animals both during the testing and subsequent scoring of the recorded behaviors. For the two batches of mice that underwent developmental milestone and UVS testing, paw tattoos were administered immediately after testing on P2, to allow for identification of pups until P14 when mice were ear notched for permanent identification (Scattoni et al, 2008). Mice tested when juvenile and adult were identified by ear notching. Conspecific mice were

housed in a separate room to the test mice to ensure the conspecifics were unfamiliar to the test mice. Test mice were never exposed to the same conspecific during testing to ensure novelty of the conspecifics.

Developmental milestones: Mice underwent a battery of tests to assess developmental milestones (Fox, 1965; Heyser, Wilson and Gold, 1995) from P2-20. Physical landmarks recorded included bodyweight and length, eyelid- and ear canal opening, fur appearance and incisor eruption. Reflexes were measured either categorically with 0 = no response and 1 = complete response or semi-quantitatively with 0 = no response, 1 = slight response, 2 = incomplete response, 3 = complete response. Reflexes measured included righting reflex, auditory- and tactile startle, rod grasping, cliff avoidance, level- and vertical screen test, negative geotaxis and reaching. (Righting reflex and negative geotaxis were measured up-to P14 only as all mice had reached this developmental milestone by this age). Locomotor behaviors were assessed categorically and those measured included head-, forelimb- and shoulder elevation and quadrupled walking. Mice were given 3 opportunities to complete the behavior, if they failed to show any response after 3 attempts, a zero value was assigned. To avoid inter-observer variability, the same experimenter recorded all developmental milestone behaviors.

Ultrasonic vocalizations (USVs): Ultrasonic vocalizations in separated pups were recorded at PND 2, 4, 6, 8 and 12. An Ultrasound Microphone (Avisoft UltraSoundGate condenser microphone capsule CM16, Avisoft Bioacoustics, Berlin, Germany), sensitive to frequencies of 10–180 kHz, was placed through a hole in the middle of the cover of the sound-attenuating box, about 20 cm above the pup in its glasses container. Vocalizations were recorded using Avisoft Recorder software (Version 3.2). For acoustical analysis, recordings were transferred to Avisoft SASLab Pro (Version 4.40) and a fast Fourier transformation (FFT) was conducted. Spectrograms were generated at a frequency resolution of 488 Hz and a time resolution of 1 ms. The number of calls was determined for each test day to define the ontogenetic profile of emission in control and conditional knockout pups, as described previously (Scattoni et al, 2008).

Open field test of anxiety: The open field test measures the conflict between a rodent's exploratory behavior and aversion to open, exposed areas (Hall, 1934). Mice

were placed facing the wall of an open field arena (40 cm dia) and allowed to freely explore the arena for 10 min. A small lamp placed on the test room floor provided dispersed lighting (25 lux). Each mouse was placed in the corner of the open field at the beginning of the trial. In Ethovision, an area of equal distance from the periphery (20 cm dia), defined as the 'central zone', was virtually drawn within the arena. The frequency of entries into, and the time spent in, the central zone of the arena were extracted in Ethovision, in addition to the total distance travelled (cm) and mean velocity (cm/ second).

Light/dark test of anxiety: The light dark box measures the conflict between rodents' exploratory behavior and aversion to open and brightly lit areas (Crawley & Goodwin, 1980). For the light/dark test, a custom-built box of white acrylic was used with dimensions (44 x 21 x 21 cm). The box was divided into two chambers by a sheet of white acrylic (21 x 50 cm); a smaller dark chamber (20 lux) that occupied roughly 1/3 of the total box, and a larger light chamber (80-110 lux) that was lit from above with a bright white light. A small doorway within a partition (5 x 7 cm) allowed the mice to move between chambers freely. Mice were placed in the dark compartment at the start of the 5-min trial. The latency (s) to enter the light chamber, time (s) spent in each chamber, and the number of light-dark transitions were measured. The mean velocity (cm/s) and total distance travelled (cm) in the dark and light compartments were extracted from the tracking software. Entry to either compartment was defined as when all four paws of the mouse had entered in one compartment.

Juvenile social investigation: Social investigations of novel, aged-matched C57BL/6J sex-matched conspecifics by juvenile (P21) control or conditional knockout mice were assessed as described previously (McFarlane et al, 2008). Mice were singly housed on P21, in a clean, standard housing cage (32×16×14cm) with sawdust (Litaspen premium, Datesand Ltd, Manchester) but no other enrichment and with *ad libitum* access to food and water. After 1h, test mice were placed into a new standard housing cage containing sawdust and a novel, juvenile (3 weeks old) sex-matched conspecific C57BL/6J mouse introduced to the test cage. The test room was dimly lit (10 lux). Mice were allowed to interact for 30 min and the behavior was recorded. The following behaviors (frequency and duration in s) initiated by the test mouse

were scored: anogenital sniffing (direct contact with the anogenital area), body sniffing (sniffing or snout contact with the flank area), head sniffing (sniffing or snout contact with the head/neck/mouth area). No observations of mounting, fighting, tail rattling, and wrestling behaviors were observed. Scoring was conducted by an experimenter blind to the genotype of the mouse.

Adult social investigation: Social investigations of novel, juvenile C57BL/6J sex-matched conspecifics by adult (>10 weeks old) control or conditional knockout mice were assessed as described previously (Grayton et al, 2013). Test mice were placed into a new standard housing cage containing sawdust and a novel, sex-matched conspecific C57BL/6J mouse introduced to the test cage. The test room was dimly lit (10 lux). Mice were allowed to interact for 5 min and the behavior was recorded. Social investigation(sniffing around the head, body and anogenital regions) initiated by the test mouse subsequently was scored from the recordings by an experimenter blind to the genotype of the mouse. No observations of mounting, fighting, tail rattling, and wrestling behaviors were observed.

3 chamber social approach task: The three-chambered social approach task assesses direct social approach behaviors when a subject mouse is presented with the choice of spending time with either a novel mouse or novel object. Sociability is defined as the subject mouse spending more time in the chamber containing the mouse than in the chamber containing the object (Yang, Silverman & Crawley, 2011). This task was carried out essentially as described by Grayton et al (2012). The mice were allowed to freely explore the three-chamber apparatus over two 10 min trials. During trial 1, the apparatus was empty and the locomotor activity (distance travelled, cm; velocity, cm/s) of, and time (s) spent in each chamber by, the mice was tracked using Ethovision. In trial 2, one wire cup containing an inanimate object (object resembled a mouse in size and shape) was placed upside down in one of the side chambers (novel object stimulus) and a novel age and sex-matched conspecific mouse was placed under another wire cup in the other side chamber (novel mouse stimulus), leaving the middle chamber empty. The location of the novel mouse across trials was counterbalanced to minimize any potential confound due to a preference for chamber location.

Olfactory habituation/dishabituation test: This test provides a control measure of intactness of animal's olfaction (Yang & Crawley, 2009). Animals were tested in their home cage, with all the enrichment removed and a fresh cage lid provided just before the trial commenced to minimize the amount of interfering odors. (The cage of each mouse was cleaned 3 days prior to testing). Following a 10 min habituation, the mouse was exposed to three odors in turn: water (control/no odor; 50µl), banana essence (non-social; 50µl; Uncle Roy's, Moffat, UK) and urine collected from novel, sex-matched conspecific mice (social, 25µl), each presented on a cotton-tipped wooden applicator 3 times over 2 minutes. Total time (s) spent by the mouse sniffing the cotton buds during each trial was recorded

Morris Water Maze (MWM): To test spatial learning and memory, the experimental animals were tested in the MWM (Morris, 1984) as described previously (Grayton et al, 2013) except mice were tested across 6 hidden platform sessions. Latency to reach the platform was manually scored for each mouse by an experiment blind to the genotype of the mouse and path length (cm) to reach the platform and speed (cm/s) were extracted from Ethovision. Mean latencies (s) and path lengths (cm) were calculated across the trials within each session for each mouse. To assess the retention of spatial memory, the time spent in the quadrant that had contained the platform (target quadrant) compared to the other quadrants was measured. Conflicting behavioral responses such as floating or thigmotaxis (the amount of time spent swimming in the outer area of the pool defined as a 15 cm wide circular zone adjacent to the wall of the maze) were assessed throughout the trials.

Grip strength: To assess the neuromuscular ability of the animals, fore- and hindlimb grip strength was measured (Whittemore et al, 2003) using a Linton Grip Strength Meter (MJS Technology Ltd, Stevenage UK). Fore- and hindlimb grip strength was measured 3 times and the mean grip strength of the 3 trials reported.

Statistical analysis. All statistical analysis was conducted using SPSS (Statistics 22 (IBM, Version Armonk, U.S.A.). Data were analysed using either a Chi-squared test (developmental milestones), Student's t-test, a 2-way ANOVA or a 2-way, repeated measures ANOVA, as appropriate. The between-factors were always sex and genotype, and within-factors either were time (olfactory habituation/dishabituation), chamber (three-chamber social approach task) or sessions (Morris water maze).

References

Brown, R.Z. (1953) Social behaviour, reproduction and population changes in the house mouse. Ecol Monogr. 23: 217-240.

Crawley J, Goodwin FK (1980) Preliminary report of a simple animal behavior model for the anxiolytic effects of benzodiazepines. Pharmacol Biochem Behav 13: 167-170.

Fox, W.M. (1965) Reflex-ontogeny and behavioral development of the mouse. Anim Behav 13: 234-241

Grayton, H.M., Missler, M., Collier, D.A., Fernandes, C (2013) Altered Social Behaviors in Neurexin 1a Knockout Mice Resemble Core Symptoms in Neurodevelopmental Disorders. PloS One 8: e67114

Hall CS (1934) Emotional behavior in the rat: Defecation and urination as measures of individual differences in emotionality. HJournal of comparative psychology 18: 19.

Harris, J. (1943) Habituatory response decrement in the intact organism. Psychological Bulletin 40:385–422.

Heyser, C.J., Wilson, M.C., Gold, L.H. (1995) Coloboma hyperactive mutant exhibits delayed neurobehavioral developmental milestones. Developmental Brain Research 89: 264-269

McFarlane, H.G., Kusek, G.K., Yang, M., Phoenix, J.L., Bolivar, V.J., Crawley, J.N. (2008) Autism-like behavioral phenotypes in BTBR T+tf/J mice. Genes Brain Behav 7:152-163.

Morris R (1984) Developments of a water-maze procedure for studying spatial learning in the rat. J Neurosci Methods 11: 47-60.

Scattoni, M.L., Gandhy, S.U., Ricceri, L. and Crawley, J.N (2008). Unusual Repertoire of Vocalizations in the BTBR T+tf/J Mouse Model of Autism. PLoS ONE 3(8): e3067

Whittemore, L.A., Song, K., Li, X., Aghajanian, J., Davies, M., Girgenrath, S., Hill, J.J., Jalenak, M., Kelley, P., Knight, A., Maylor, R., O'Hara, D., Pearson, A., Quazi,

A., Ryerson, S., Tan, X.Y., Tomkinson, K.N., Veldman, G.M., Widom, A., Wright, J.F., Wudyka, S., Zhao, L., Wolfman, N.M. (2003) Inhibition of myostatin in adult mice increases skeletal muscle mass and strength. Biochem Biophys Res Commun 300: 965-971

Yang M, Crawley JN (2009) Simple behavioral assessment of mouse olfaction. Curr Protoc Neurosci Chapter 8: Unit 8 24.

Yang M, Silverman JL, Crawley JN (2011) Automated three-chambered social approach task for mice. Curr Protoc Neurosci Chapter 8: Unit 8 26.

Supplementary Table 1. Reduced postnatal survival of *Nestincre;Chd7cko* animals

Cross	Stage	Expected frequency of cko	Observed frequency of cko	Chi Square	P value
Nestincre;$Chd7^{f/+}$* x *$Chd7^{f/f}$	P0	¼ (25%)	3/13 (23%)	0.019	0.8897
	P2	¼ (25%)	2/6 (33%)	0.167	0.6831
	P14	¼ (25%)	**1/8 (12.5%)**	0.500	0.4795
	P21	¼ (25%)	**2/34 (0.06%)**	6.627	**0.01 ****

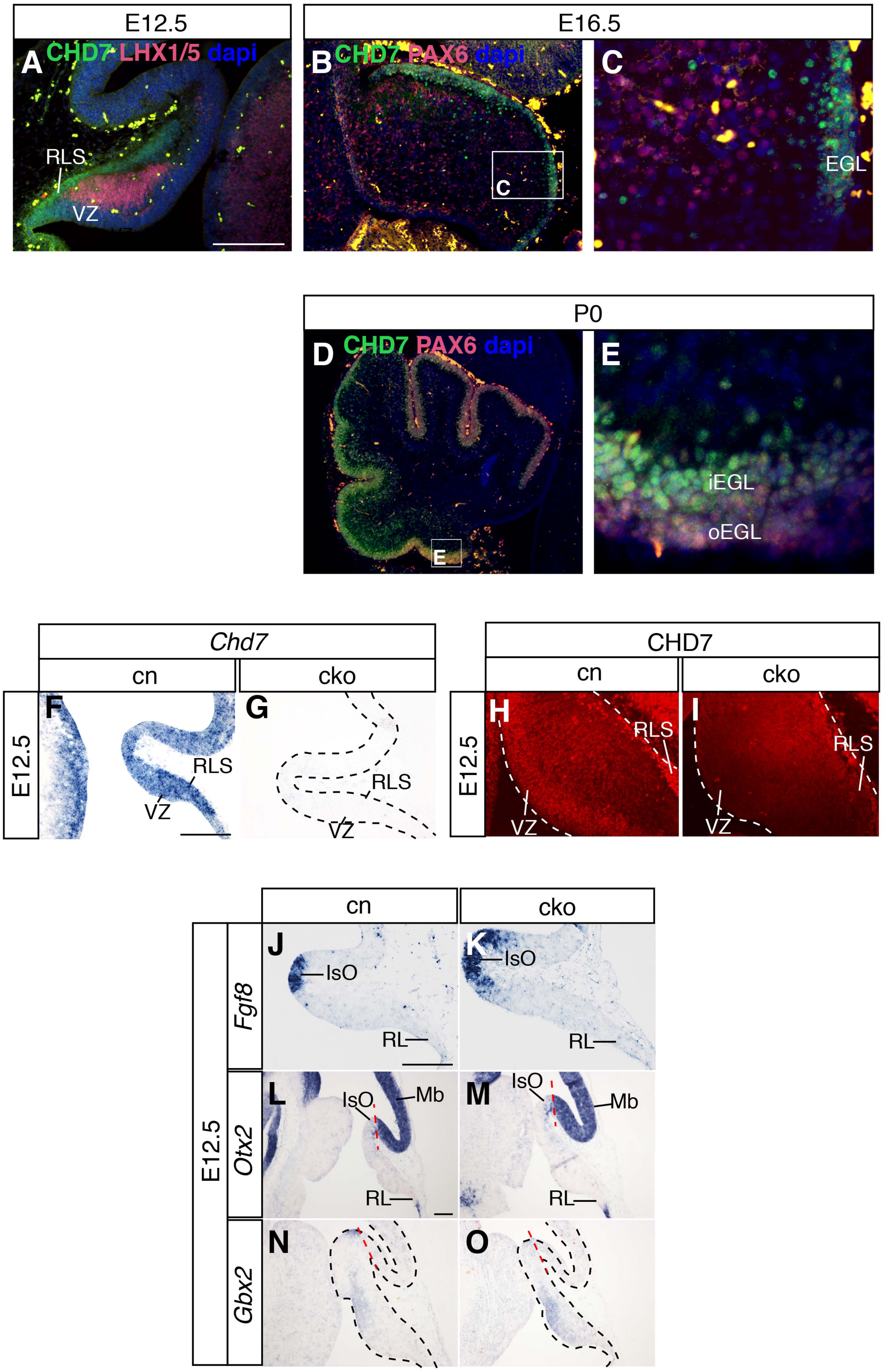

Supplementary Figure 1. *Chd7* is efficiently deleted from neuronal progenitors of *Nestincre;Chd7$^{f/f}$* mice without disruption of the isthmic organizer. (A-E) Immunohistochemistry of sagittal sections through the cerebellum at E12.5 (A), E16.5 (B,C) and P0 (D,E) using antibodies to CHD7, LHX1/5 and PAX6 as indicated. CHD7 is detected predominantly in derivatives of the rhombic lip (A-E). CHD7 protein is present in the EGL from embryonic stages (B,C), and is increased in cells in the inner EGL (iEGL) at P0. (F,G) In situ hybridization of sagittal sections through the cerebellum at E12.5 of *Chd7$^{f/f}$* (cn) and *Nestincre;Chd7$^{f/f}$* (cko) mice using a *Chd7* antisense probe to exon 3, anterior to the top. The dorsal neural tube is demarcated by a black dashed line (G). (H,I) Immunohistochemistry of sagittal sections through the cerebellum at E12.5 using an anti-CHD7 antibody. The ventricular zone (VZ) and the rhombic lip stream (RLS) are indicated. (J-O) In situ hybridization to detect *Fgf8* (J,K), *Otx2* (L,M) and *Gbx2* (N,O) transcripts in sagittal sections through the cerebellum and midbrain (J-O), anterior to the top. Note similar levels of *Fgf8* expression in the isthmic organiser of the cko compared to the cn. Note that the position of *Otx2* and *Gbx2* expression remains unaltered in the cko compared to the cn as indicated by a red dashed line (L-O). RL= rhombic lip, RLS= rhombic lip stream, VZ= ventricular zone, IsO= Isthmic organiser, Mb=midbrain. Scale bar= 300µm (F,J,L).

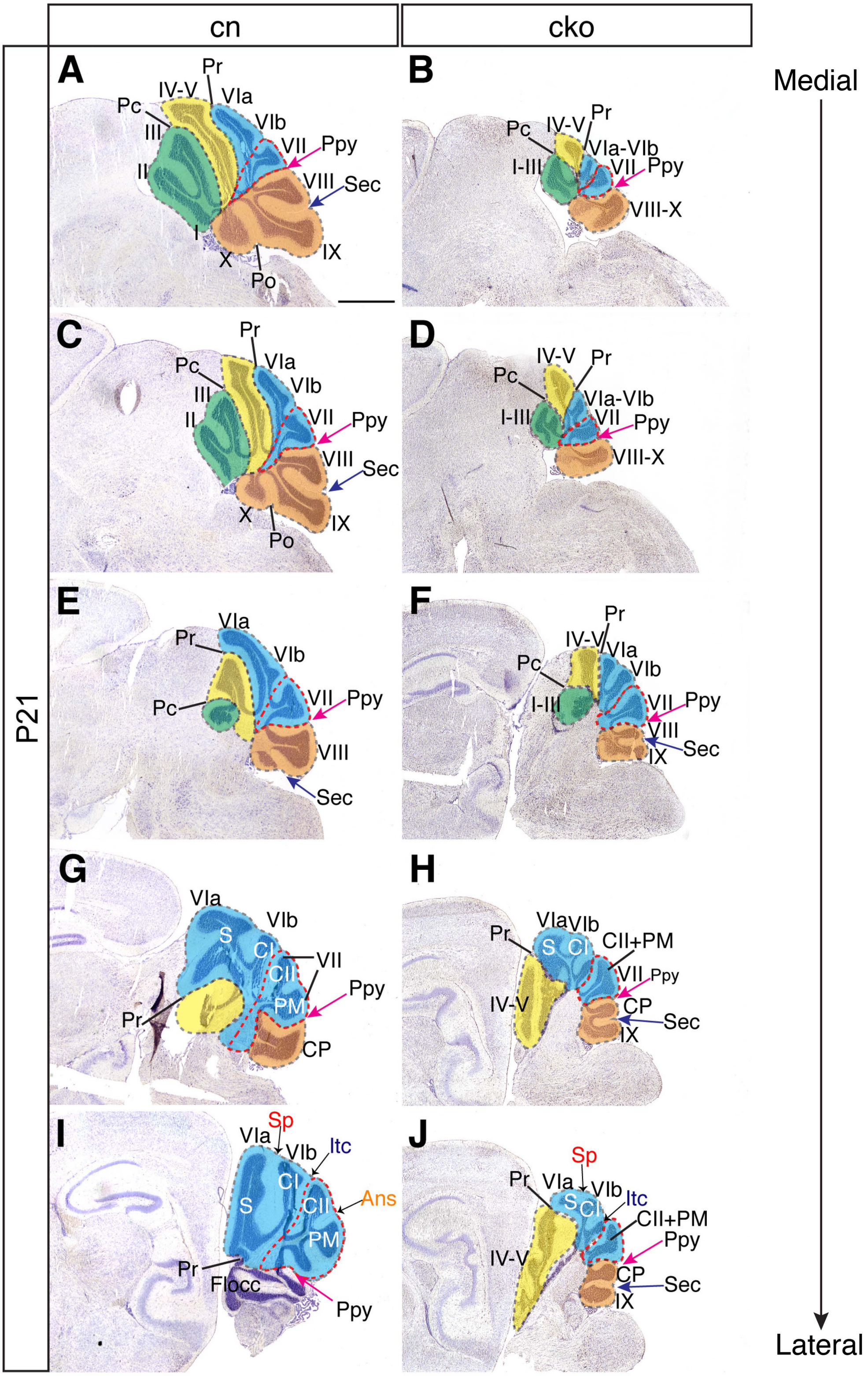

Supplementary Figure 2. Cerebellar foliation abnormalities in *Nestincre;Chd7$^{f/f}$* (cko) mice

(A-J) Cresyl violet-stained sagittal sections through the cerebella of *Chd7$^{f/f}$* (cn) and *Nestincre;Chd7$^{f/f}$* (cko) mice, anterior to the left. Representative sections along the medial to lateral extent of the cerebella are shown. Folia in the vermis are labelled with Roman numerals. The Simplex (S), CrusI (CI), CrusII (CII) and paramedian (PM) folia in the hemispheres are labelled. Shading indicates I-III (green), IV-V (yellow), VI-VII (blue), VIII-IX (orange). Lobules are labelled according to their lobule of origin in the vermis. Note that foliation is severely disrupted throughout the mediolateral axis with anterior and posterior vermis-specific lobules extending into the hemispheres in the cko cerebellum. Pc= preculminate, Pr= primary, Ppy= prepyramidal, Sec= secondary, Po= posterolateral, Sp= superior posterior, Itc= intercrural, Ans= ansoparamedian, Flocc= Flocculus. Scale bar= 1mm (A).

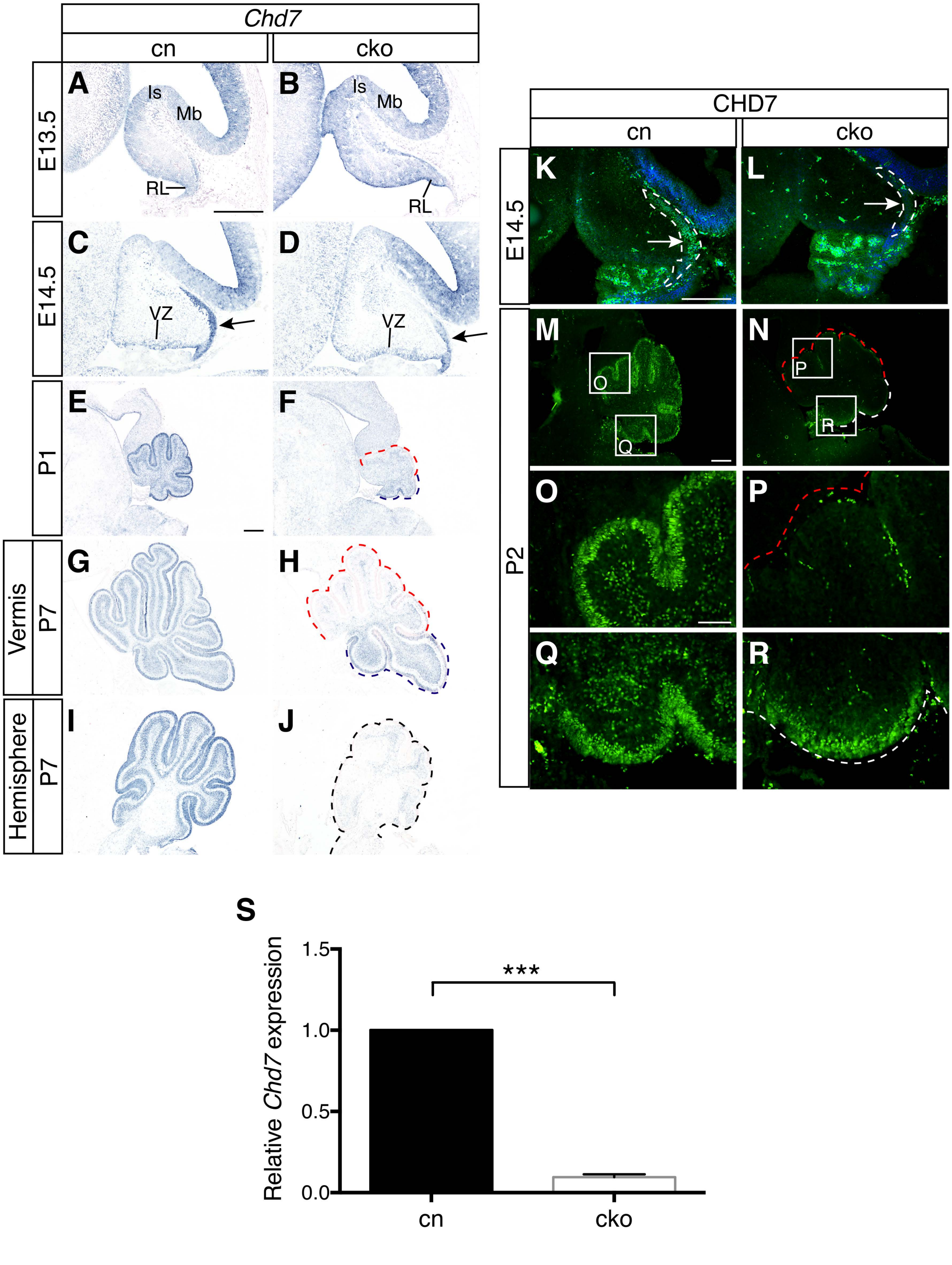

Supplementary Figure 3. Efficient *Chd7* deletion from the cerebellar granule cells of *Math1cre;Chd7f/f* mice. (A-J) In situ hybridization on sagittal sections from the developing cerebellum at the indicated stages, anterior to the left, using an antisense probe to exon 3 of the *Chd7* gene. (K- R) Immunohistochemistry of sagittal sections through the cerebellum at indicated stages using an anti-CHD7 antibody. The EGL and rhombic lip are demarcated by a white dashed line (K,L). (A-B) Note a similar level of *Chd7* expression in the cko cerebella compared to the cn at E13.5. Successful recombination of the $Chd7^{flox}$ allele by the *Math1cre* transgene in the anterior EGL from E14.5 is indicated by an arrow (D,L). Note the absence of *Chd7* in the granule cells of the anterior vermis (lobules I-VIII) by in situ hybridization and immunohistochemistry (F,H,N,P) indicated by a red dashed line, with *Chd7* expression retained in the posterior lobules (IX+X) indicated by a navy (F,H) and white (N,R) dashed line. Note complete absence of *Chd7* in the granule cells of the cerebellar hemispheres indicated by a black dashed line (J). (S) Quantification of *Chd7* transcripts in purified P7 GCps by qRT-PCR. Note the reduction of *Chd7* in cko cells, relative to cn cells (Data from 3 independent samples of purified GCps per genotype). ***$P<0.001$, Student's T-test. Scale bar= 300µm (A,E,K,M), 100µm (O).

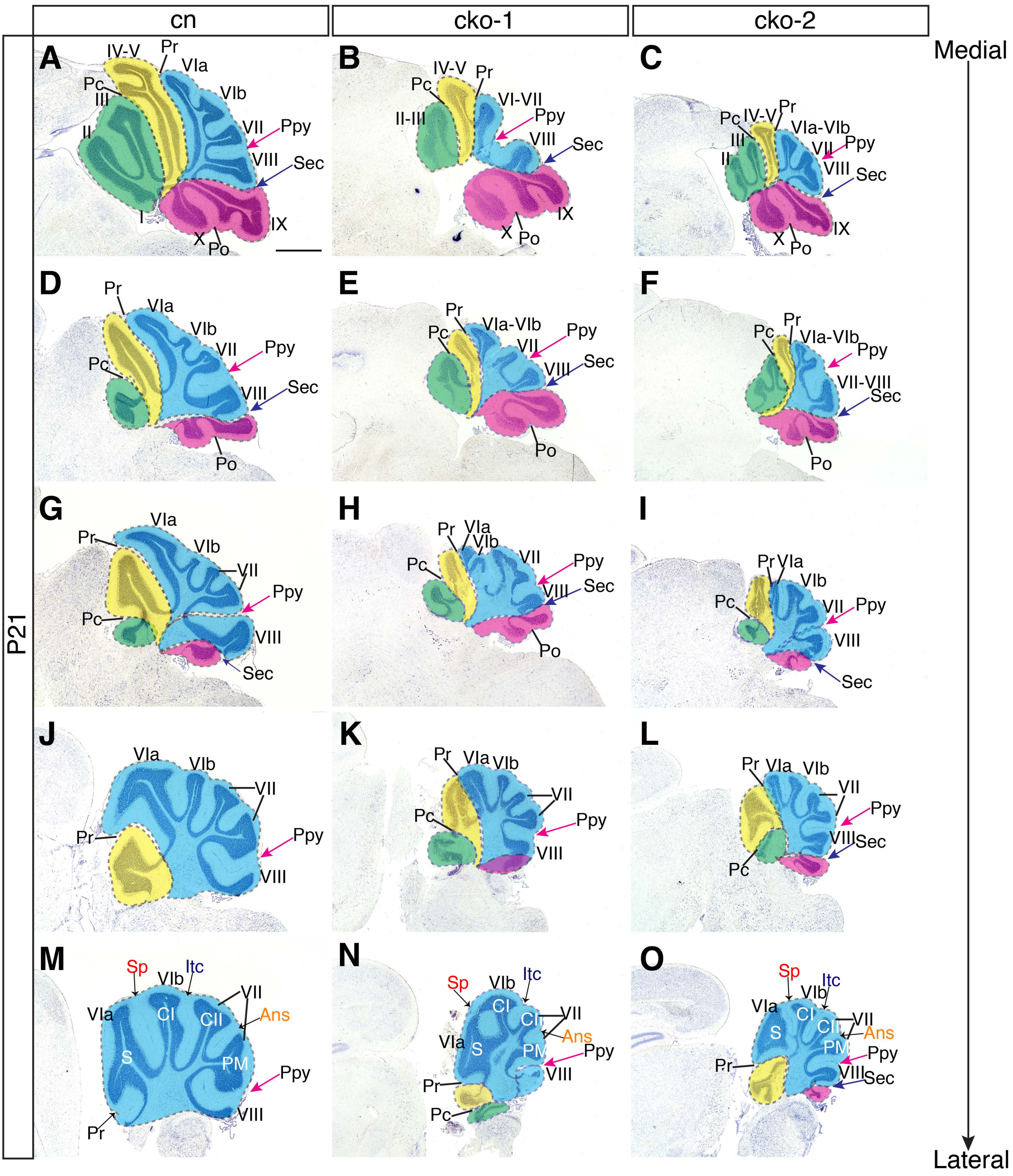

Supplementary Figure 4. Deletion of *Chd7* from cerebellar GCps disrupts foliation across the entire mediolateral axis.

(A-O) Cresyl violet stained sagittal sections through the cerebellum of a cn and two examples of cko (cko-1, cko-2) mice. Representative sections along the medial to lateral extent of cerebella are shown. Folia in the vermis are labelled with Roman numerals. The Simplex (S), CrusI (CI), CrusII (CII) and paramedian (PM) folia in the hemispheres are labelled. Green shading indicates lobules I-III, yellow indicates IV-V, blue indicates VI-VIII and pink indicates IX and X. Note the extension of the anterior and posterior vermis-specific lobules into the hemispheres of cko-1 and cko-2. Pc= preculminate, Pr= primary, Ppy= prepyramidal, Sec= secondary, Po= posterolateral, Sp= superior posterior, Itc= intercrural, Ans= ansoparamedian. Scale bar= 1mm (A).

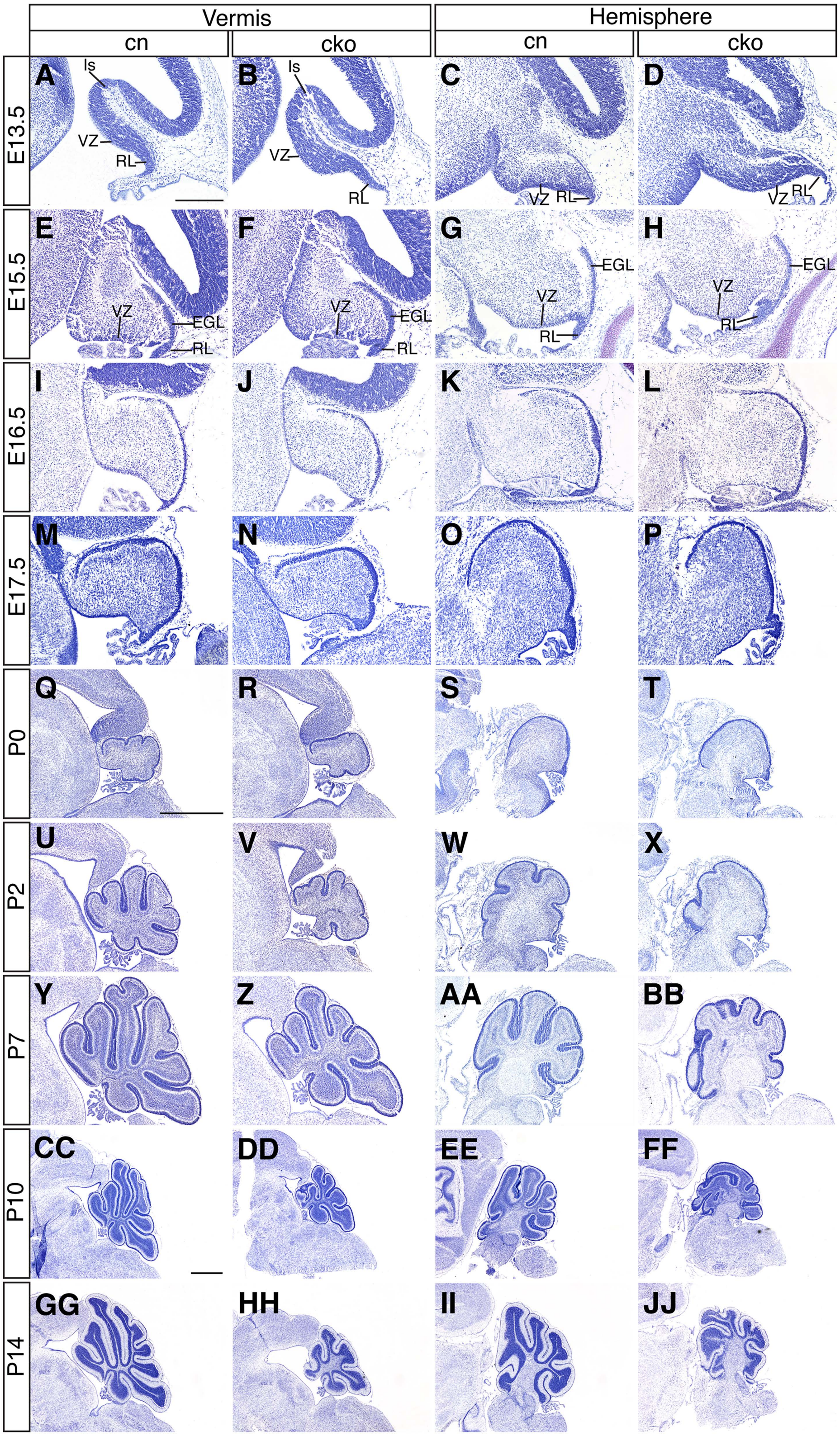

Supplementary Figure 5. Time course of cerebellar development in GCp-specific mutants demonstrate the perinatal appearance of cerebellar hypoplasia and foliation defects. (A-JJ) Cresyl violet-stained sagittal sections through the cerebellar vermis and hemispheres of cn and cko brains at the indicated developmental stages are shown. Note the first signs of abnormal cerebellar development becoming evident at E17.5 in the cerebellar vermis with clear hypoplasia noted at P0. Subtle changes in foliation appear in hemispheres at P2, with hypoplasia and foliation abnormalities clearly evident at P7. Is = isthmus organizer, RL = rhombic lip, VZ = ventricular zone, EGL = external germinal layer. Scale bar= 300µm (A), 1mm (Q, CC).

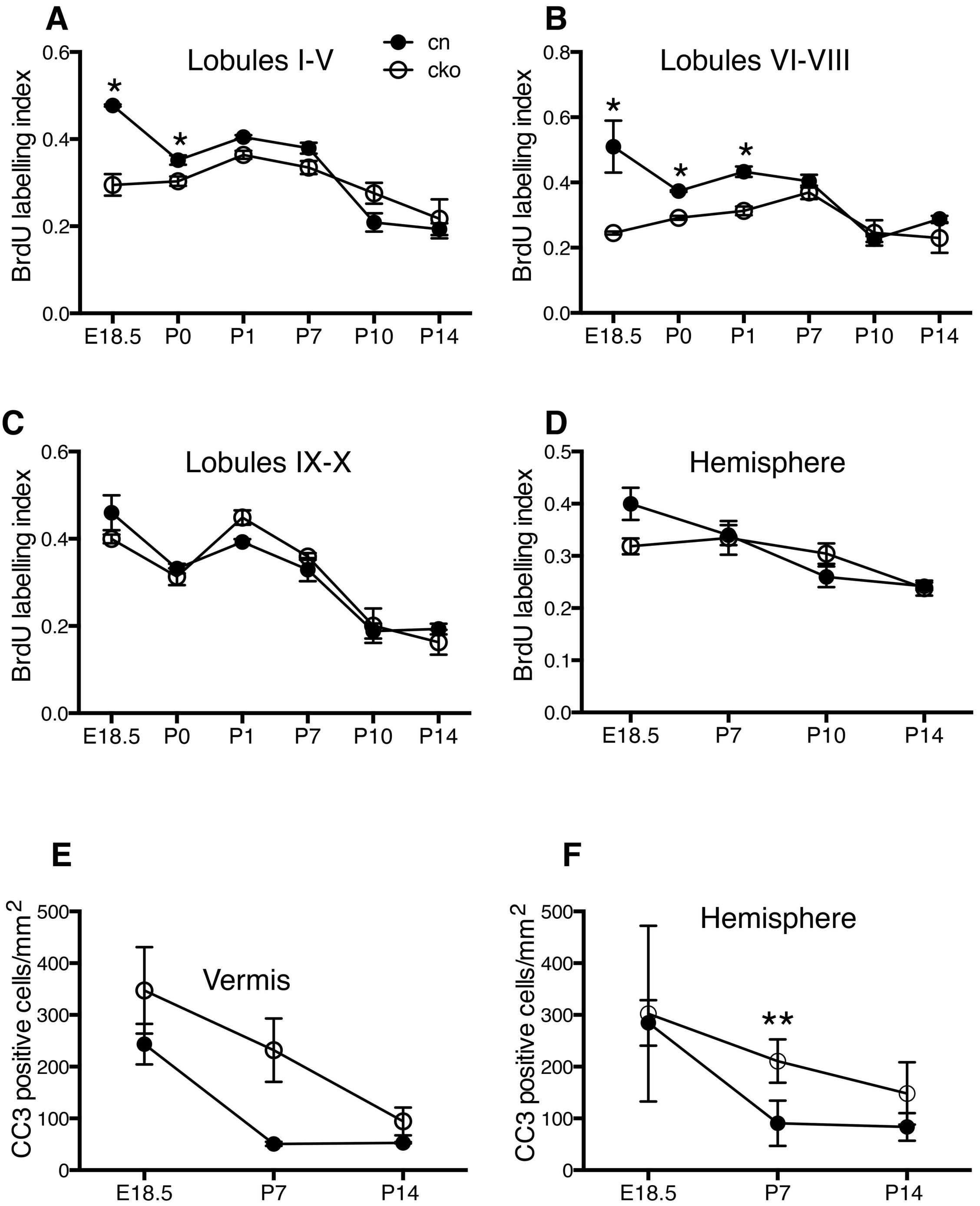

Supplementary Figure 6. CHD7 regulates GCp proliferation and apoptosis in the perinatal cerebellum, with different dynamics in the vermis and hemispheres. (A-D) Quantification of the BrdU labelling index of cn and cko GCps in regions and at stages indicated. GCp proliferation was significantly reduced in the anterior (I-V) and central lobules (VI-VIII) of the cko mice in the perinatal period. No significant difference in proliferation was detected in lobules IX-X and the hemispheres at any stage ($n \geq 3$ per genotype). (E-F) Quantification of the total number of cleaved caspase-3 (CC3) positive cells in the EGL/ mm^2 of cerebellar GCps in the vermis and hemispheres at stages indicated. Note significantly increased apoptosis in the hemispheres of the cko at postnatal day 7 (F) (n=3 per genotype). *$P<0.05$, Student's T test.

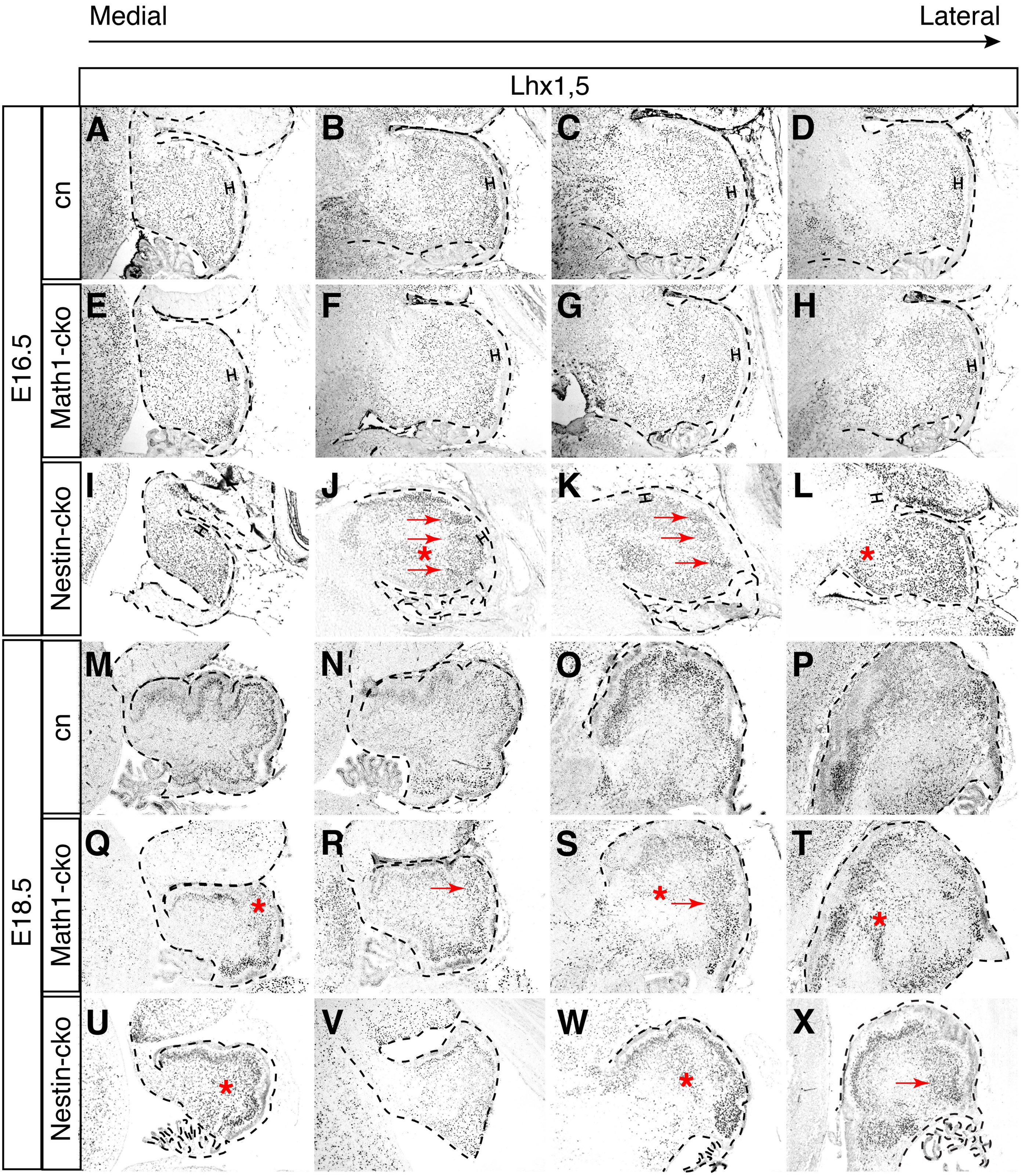

Supplementary Figure 7. CHD7 is necessary for normal Purkinje cell distribution.

(A-X) Lhx1,5 immunohistochemistry on sagittal sections through the cerebellum to label PCs at E16.5 (A-L) and E18.5 (M-X), anterior to the left. Note the presence of the PC plate in the vermis of cn, and Math1-cko and Nestin-cko mice at E16.5 (brackets). Clusters of disorganized PCs in the centre of the cerebellum are indicated by asterisks, and abnormal PC distributions under the EGL by red arrows. Note abnormalities at E16.5 and E18.5 in the Nestin-cko and at E18.5 in the Math1-cko. Scale bar= 300µm (A,I), 100µm (E,M,Q).

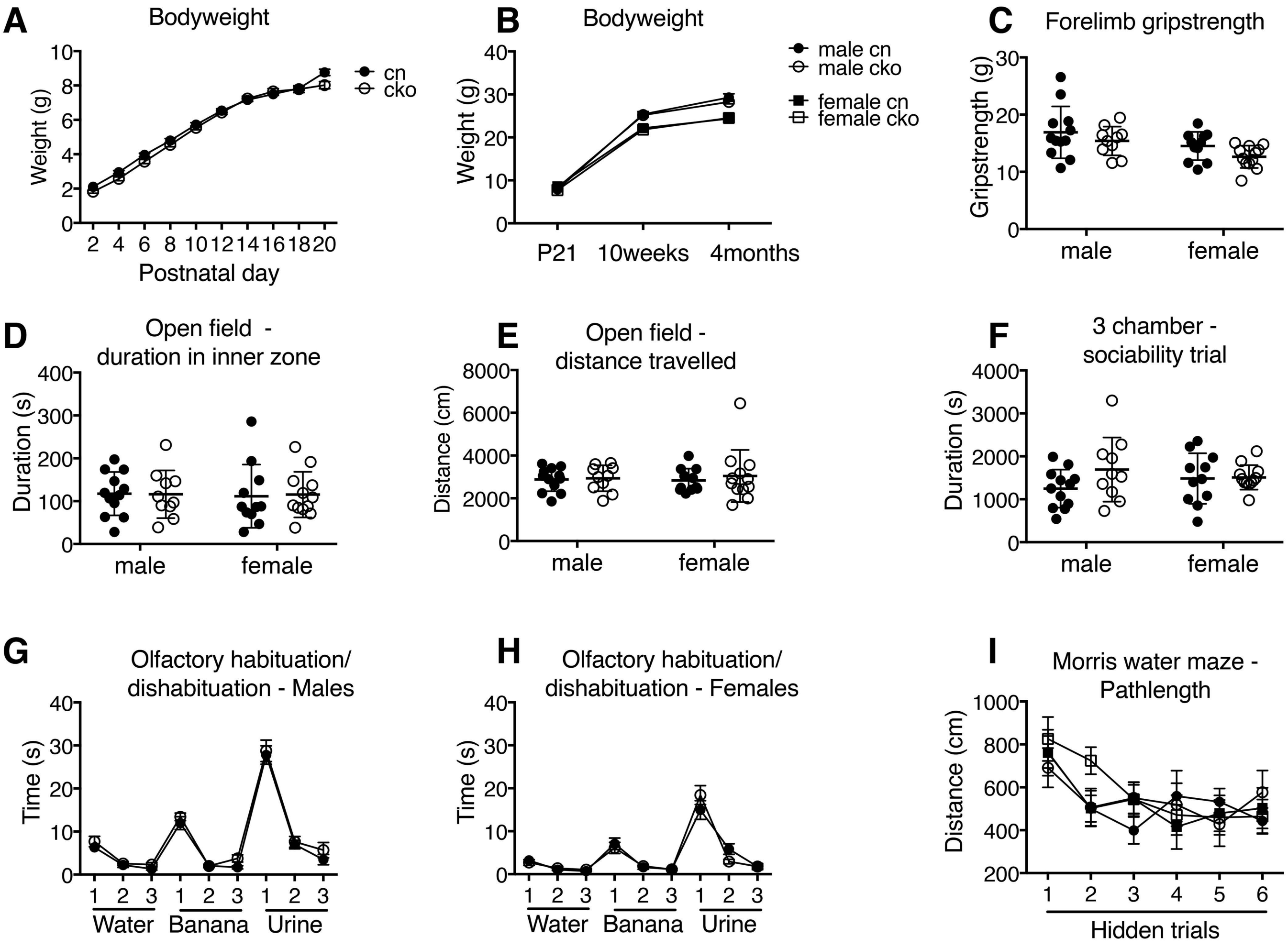

Supplementary Figure 8. Additional behavioral analyses. Behavioral assessments of a cohort of adult (B-I) cn (male n=12, female n=11) and cko (male n=10, female n=12) mice, and a cohort of pups for analysis of developmental milestones(A) (cn n=20, cko n=22). (A,B) Mean bodyweight for the developmental milestones test batch (A) and the adult test batch (B) at indicated ages. No significant differences in bodyweight were found (repeated-measures ANOVA, with Student's T-test as post-hoc analysis). (C) Average forelimb grip strength. No significant difference between the cn and the cko groups were seen (between-subjects ANOVA). (D) Graph plotting the time spent in the centre of the open field during a 10 minute test. No significant difference between the cn and the cko groups (between-subjects ANOVA). (E) The total distance travelled in the open field during a 10 minute test. No significant difference was seen between the cn and the cko groups (between-subjects ANOVA). (F) The duration, in seconds, spent in the chamber with the unfamiliar, conspecific mouse. No significant difference between cko and cn mice. (between-subject ANOVA). (G,H) Graph demonstrating the performance of male (G) and female (H) animals on the olfactory habituation/dishabituation test. No significant differences between the groups were found (repeated-measures ANOVA). (I) Graph plotting the average distance swum by cn and cko animals for 4 trials daily over 6 consecutive training days to find the hidden platform. No significant difference in performance was seen between the groups (repeated-measures ANOVA).
